# Mitochondrial C3a Receptor Activation in Oxidatively Stressed Epithelial Cells Reduces Mitochondrial Respiration and Metabolism

**DOI:** 10.3389/fimmu.2021.628062

**Published:** 2021-03-05

**Authors:** Masaaki Ishii, Gyda Beeson, Craig Beeson, Bärbel Rohrer

**Affiliations:** ^1^Department of Ophthalmology, Medical University of South Carolina, Charleston, SC, United States; ^2^Department of Drug Discovery and Biomedical Sciences, Medical University of South Carolina, Charleston, SC, United States; ^3^Ralph H. Johnson Veterans Affairs Medical Center, Charleston, SC, United States; ^4^Department of Neurosciences, Medical University of South Carolina, Charleston, SC, United States

**Keywords:** mitochondria, complement C3a receptor, translocation, endosomal targeting, calcium imaging, oxidative phosphorylation

## Abstract

Complement component 3 fragment C3a is an anaphylatoxin involved in promoting cellular responses important in immune response and host defense. Its receptor (C3a receptor, C3aR) is distributed on the plasma membrane; however, lysosomal localization in immune cells has been reported. Oxidative stress increases intracellular reactive oxygen species (ROS), and ROS activate complement signaling in immune cells and metabolic reprogramming. Here we tested oxidative stress and intracellular complement in mitochondrial dysfunction in RPE cells using high resolution live-cell imaging, and metabolism analysis in isolated mitochondria using Seahorse technology. While C3aR levels were unaffected by oxidative stress, its cell membrane levels decreased and mitochondrial (mt) localization increased. Trafficking was dependent on endocytosis, utilizing endosomal-to-mitochondrial cargo transfer. H_2_O_2_-treatment also increased C3a-mtC3aR co-localization dose-dependently. In isolated mitochondria from H_2_O_2_-treated cells C3a increased mitochondrial Ca^2+^ uptake, that could be inhibited by C3aR antagonism (SB290157), mitochondrial Ca^2+^ uniporter blocker (Ru360), and Gαi-protein inhibition (pertussis toxin, PTX); and inhibited mitochondrial repiration in an SB290157- and PTX-dependent manner. Specifically, mtC3aR activation inhibited state III ADP-driven respiration and maximal respiratory capacity. Mitochondria from control cells did not respond to C3a. Furthermore, transmitochondrial cybrid ARPE-19 cells harboring J haplogroup mitochondria that confer risk for age-related macular degeneration, showed high levels of mtC3aR and reduced ATP production upon C3a stimulation. Our findings suggest that oxidative stress increases mtC3aR, leading to altered mitochondrial calcium uptake and ATP production. These studies will have important implication in our understanding on the balance of extra- and intracellular complement signaling in controlling cellular health and dysfunction.

## Introduction

The complement system is an essential part of the innate and adaptive immune system to eliminate foreign antigens and pathogens as part of the normal host response (1). The complement system is activated by various types of stimuli, such as oxidative stress, inflammatory factors, and ischemia (2). Depending on the pathogen-associated molecular patterns (PAMPs) or damage-associated molecular patterns (DAMPs), the complement system can activate 3 distinct pathways: the classical pathway (CP), lectin pathway (LP), and alternative pathway (AP). The 3 pathways all participate in the formation of C3 convertases that will catalyze the proteolytic cleavage of complement component 3 (C3) into C3a and C3b. C3b subsequently participates in the formation of a C5 convertase that cleaves complement component 5 (C5) into C5a and C5b. as the final step of the complement cascade, C5b initiates in the formation of the membrane attack complex (MAC) on membranes together with C6, C7, C8, and C9 to promote sublytic cell signaling or cell lysis (3). Anaphylatoxins have multiple roles, including vasodilation and enhanced vascular permeability, as well as the mediation of chemotaxis and inflammation, and the generation of cytotoxic oxygen radicals (4), which are mediated by their receptors C3aR, C5aR, and C5L2 (5).

C3aR, C5aR, and C5L2 are members of the superfamily of 7 transmembrane spanning G protein-coupled receptors (GPCRs). The C3a receptor is expressed on a wide range of cell types and has mostly been studied in cells of myeloid origin including monocyte/macrophages and microglia (4). Additionally, tissues including lung, liver, kidney, brain, heart, muscle, and testis have been shown to express C3aR, in particular endothelial and epithelial cells (4). Finally, based on the involvement of the complement system in the disease of age-related macular degeneration (6), anaphylatoxin receptor expression and signaling has also been investigated in retinal pigment epithelial cells (RPE), which form part of the outer blood retina barrier of the eye and are a main target of complement activation (7).

The main recognized function of the anaphylatoxins is to translate the information about danger from the fluid phase into appropriate cellular responses. Thus, not surprising, most of the anaphylatoxin receptors are localized to the plasma membrane. However, recently intracellular anaphylatoxin receptor signaling has been documented in T-cells, including the engagement of intracellular C5aR resulting in the generation of mitochondrial ROS and C3aR signaling on lysosomes (8). In recent years, several GPCRs thought to be present only on the plasma membrane have also been identified on mitochondria. Those include the cannabinoid CB1R (9), the serotonin 5HT4R (10), the melatonin receptor MT1 (11), and the GABA_B_ receptor (12). These receptors are localized on mitochondria constitutively and contribute to normal cell physiology.

Here, we made the surprising discovery that C3aR localizes to mitochondria (mtC3aR) in oxidatively stressed H_2_O_2_-treated human retinal pigment epithelial cells (ARPE-19 cells). Furthermore, we proved that plasma membrane C3aR was internalized and transported to mitochondria by endosomal trafficking. In isolated mitochondria, we demonstrated that mtC3aR activation elevated mitochondrial Ca^2+^ level and inhibited respiratory function via the mtC3aR-G-protein signal transduction cascade. Finally, in transmitochondrial ARPE-19 cybrids with known decreased ATP production, we discovered higher levels of C3aR localization on mitochondria and mtC3aR mediated effects on ATP production even in the absence of exogenous oxidative stress. These studies will have important implication in epithelial cell biology and the future development of anti-complement therapeutics, as they will contribute to our understanding on the balance of extra- and intracellular complement signaling in controlling cellular health and dysfunction.

## Materials and Methods

### Cell Lines

ARPE-19 cells (immortalized human RPE cells, passage 22–40; ATCC, Manassas VA), expanded in DMEM including 10% fetal bovine serum (FBS) with 1% antibiotic–antifungal agents (Thermo Fisher, Waltham MA), were chosen, as, when grown as monolayers, they express all the signature genes of human RPE cells, develop tight, adherence and gap junctions, and resemble an aged RPE over time (13, 14).

Transmitochondrial APRE-19 cybrids cells from the H- or the J-haplogroup were generously provided by Cris Kenney (University of Irvine, Irvine CA) (15–17). Cybrids were grown in DMEM/Ham's F12 1:1 (Invitrogen-Gibco, Gaithersburg MD) medium containing 24 mM NaHCO3, 10% dialyzed FBS, and 1.0 mM C_3_H_3_NaO_3_.

As positive and negative controls for C3aR expression, HeLa (human cervical epithelial cells, CCL-2TM, ATCC) and HEK293 (human embryonic kidney cells, CRL-1573TM, ATCC) cells were used, respectively. Both cell lines were grown in Eagle's Minimum Essential Medium and 10% FBS according to ATCC's recommendations.

### Mitochondrial Isolation

We previously developed a mitochondrial isolation method for ARPE-19 cells which yield calcium-free mitochondria for analysis as described in Ishii et al. (18). The final mitochondrial pellet was collected and resuspended in the appropriate buffers: Mitochondrial Assay Solution buffer for XFe96 respirometric assays and live cell imaging experiment or RIPA buffer for protein extraction. The same technique was utilized for the isolation of mitochondria from HeLa and HEK293 cells.

### Immunocytochemistry

Immunocytochemistry was performed on cells grown for >2 weeks on glass bottom dishes. Cells were labeled live with fluorescence dyes, MitoTracker Deep Red (MTDR, Molecular Probes, Eugene OR), and Hoechst33342 (Thermo Fisher), followed by fixation in 4% paraformaldehyde. After PBS washes, cells were incubated with antibodies against C3a receptor (clone 17, Santa Cruz, Dallas TX; for confirmation in Supplementary Figures, clone 12, Santa Cruz, was utilized) and/or C3a (rabbit anti-human C3a, Complement Technology, Tyler TX). Localization was detected using Alexa Fluor 488/564 secondary antibodies (Invitrogen, Carlsbad CA).

### Western Blotting

Cells or isolated mitochondria were homogenized in RIPA buffer supplemented with protease inhibitor cocktail and 10 mM phenylmethylsulfonyl fluoride (Sigma-Aldrich, St. Louis MO) and phosphorylation inhibitor (Roche, Sigma-Aldrich). Cell homogenates were subsequently centrifuged (800 x *g* for 5 min) to separate nucleus and supernatant, and cytosolic proteins were purified (Subcellular protein Fraction kit; Thermo Scientific). 20/30 μg protein measured by NanoDrop (Thermo Scientific) was loaded per well. Following separation, protein was transferred to PVDF membranes (iBlot system; Invitrogen), and probed with anti-C3a and anti-C3a receptor antibodies (see Immunohistochemistry) at 1:500, followed by HRP-conjugated secondary IgG antibodies (South Dakota Biotech, Falls SD). Chemiluminescent substrate for HRP (Pierce ECL Western Blotting Substrate; Thermo Scientific) was utilized, signal captured (ImageQuant LAS 4000 gel imaging system; GE Healthcare Bio-Sciences, Pittsburgh PA) and analyzed with ImageJ software (NIH, Bethesda MD). Blots were normalized for loading differences with β-actin (Santa Cruz) for cellular proteins or Cox IV for mitochondrial proteins (Abcam, Cambridge MA).

### Transfection With C3aR-GFP, Rab7a-RFP, and Lamp1-RFP Plasmids

Plasmids for C3aR-GFP (human C3a Receptor cDNA ORF Clone, C-GFPSpark^®^, tag; Sino Biological, Wayne PA), RFP-Rab7 [canine Rab7, N-terminal fusion with red fluorescent protein, RFP; Addgene, Watertown MA; (19) and Lamp1-RFP [rat Lamp1, C-terminal fusion with RFP; Addgene (20) were used for transient transfections. ARPE-19 cells were seeded at 70% confluency in 10% FBS containing DMEM on 96-well-glass bottom plates, followed by transfection (Lipofectamine 3000; Thermo Fisher), mixing 0.5 μg plamid DNAs with P3000 reagent. Cells were used for experiments after 2–3 days.

### C3a ELISA

To measure the release of C3a from cells, a Complement human C3a ELISA kit (Invitrogen) was used. Supernatants from a single 12-well-plate were precleared (14,000 × g for 5 min) and 50 μl used for analysis. Captured C3a was detected using a C3a-specific antibody conjugated with biotin, followed by signal detection by streptoavidin conjugated to HRP. Values were compared to a C3a standard.

### Live Cell Imaging

For imaging, medium was replaced to phenol red free DMEM (Thermo Fisher), with 20 mM HEPES (pH 7.3–7.4). Live cell imaging of C3aR-GFP transfected cell was carried out on glass bottom 96-well-plates (Mattek, Ashland, MA), with cells labeled with plasma membrane dye Wheat Germ Agglutinin (WGA) Deep Red (5 μg/mL; Thermo Fisher), mitochondrial maker MTDR (1 μM; Thermo Fisher) and nuclear marker Hoechst33342 (0.5 μg/mL; Thermo Fisher). Image acquisitions were performed using the UltraViewVoX spinning disk confocal microscope (Eclipse Ti, Nikon, Tokyo JPN), running Volocity software (Perkin Elmer, Wokingham UK) as published (21). Temperature and cellular pH were maintained using a table top incubator. Images were processed using ImageJ software (NIH). Some chemicals, dynasore, a dynamin inhibitor (200 μM; Sigma-Aldrich,), MnTBAP chloride, a superoxide dismutase mimetic (100 μM; Sigma-Aldrich) and CID1067700, an inhibitor of nucleotide binding by Ras-related GTPases (= Rab7 inhibitor; 100 nM; Sigma-Aldrich) were applied.

### Imaging of Isolated Mitochondria

Prior to mitochondria isolation, cells were incubated in phenol free DMEM containing 25 mM HEPES, with/without 0.5 mM H_2_O_2_, followed by labeling with MTDR and calcium indicator dye Fluo-8 AM (AAT Bioquest, Sunnyvale CA). Mitochondria were then isolated as described above. Mitochondria resuspended in MAS1X buffer were plated on 96-well-glass bottom plate by centrifugation (4,700 × g, 20 min). Some chemicals, C3a (260 nM; Complement Technology Inc, Tyler TX), C3aR antagonist SB290157 (300 nM; EMD Millipore, Burlington MA), heterotrimeric Gi/o proteins inhibitor Pertussis toxin (PTX; 1 μg/mL; Tocris), mitochondrial permeability transition pore blocker cyclosporine A (1 nM; Sigma-Aldrich) and a selective inhibitor of the mitochondrial calcium uptake system Ru360 (10 nM; EMD Millipore) were applied before recording. 10 μM CaCl_2_ was added to the medium to trigger Ca^2+^ uptake. Fluorescence was acquired at 1 s interval stimulating with both the 488 and 640 nm excitation laser. The fluorescence intensity wave data were analyzed using Igor Pro wave analysis software (WaveMetrics, Portland OR).

### Image Analysis

All confocal images in a Z-stack per channel were converted individually into tif files and processed using ImageJ software (NIH). Colocalized particles based on X–Y coordinates were detected with the Colocalization plugin and quantified with the Particle analyzer module in ImageJ as published previously (22).

### High Resolution Respirometric Assay for Isolated Mitochondria

The method of high resolution respirometric assay in isolated mitochondria using the XFe96 Seahorse assay system (XF assays, Agilent Technologies, Santa Clara CA) was described in (18). Specifically, some assays were performed in the presence of PTX (1 μg/mL) or bicarbonate, ${\text{HCO}}_{3}^{-}$, a simulator of soluble adenylate cyclase (5 mN, Thermo Fisher). The assays were separated into 2 procedures as mitochondria have a limited time to support optimal respiration upon isolation: (1) injection of ADP to produce state III respiration (ADP drive), and (2) injection of the protonophore carbonyl cyanide-p-trifluoromethoxyphenylhydrazone (FCCP; 1 μM; Tocris) to measure maximum uncoupled respiration (FCCP response). Data in each well was normalized with the value of the mitochondrial coverage measured from phase contrast images (Incucyte Zoom; Essen Bioscience, Ann Arbor Ml) and analyzed using the XFe96 Wave software (Agilent Technologies). Data from 9 to 25 wells in 3–5 independent experiments are reported.

### Data Analysis

For live-cell imaging, n represents the number of independent imaging sessions, for immunohistochemistry, ELISA, western blotting and XF assays, the number of wells examined. Results were analyzed using GraphPad Prism Statistical analysis software (version 8.1; La Jolla, LA, USA) and SPSS statistics software (version 25; IBM, Chicago IL). Data are reported as the mean ± SEM. Statistical significance was determined for all conditions of an experiment using a one-way ANOVA (*n* > 3 per group) or a Kruskal–Wallis *H*-test (*n* = 3 per group); if significance was achieved for the group comparison (*P* < 0.05), individual comparisons were performed using Student's *T*-tests or Mann–Whitney *U*-Test, respectively.

## Results

### Mitochondrial C3aR Localization Increased by Oxidative Stress

**Figure 1:**
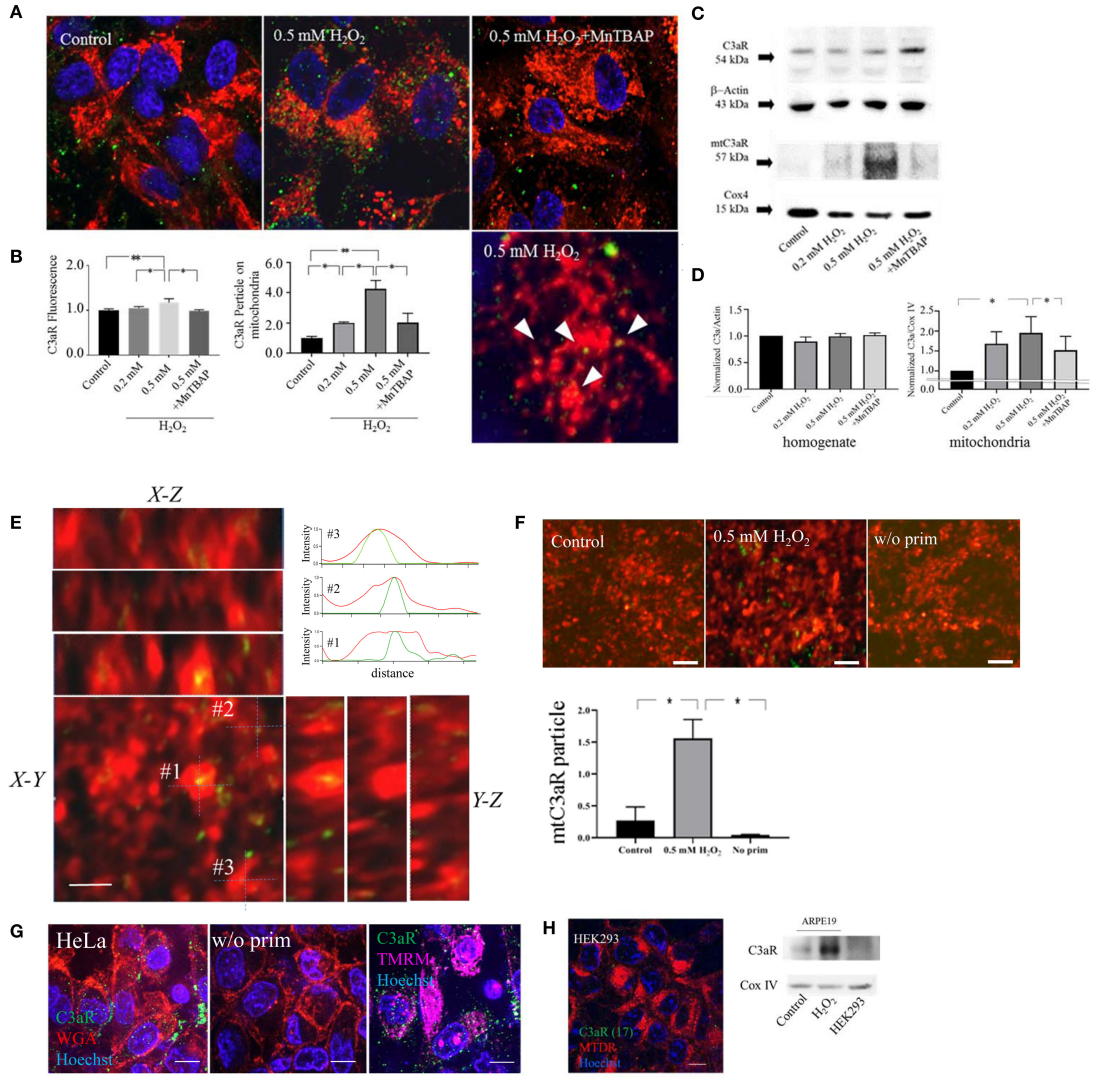
C3aR localization on mitochondria is increased by oxidative stress. Confocal imaging of C3aR immunolabeling **(A)** and western blot analysis **(B)** was performed on H_2_O_2_-treated ARPE-19 cell and isolated mitochondria. **(A)** ARPE-19 cells were treated with H_2_O_2_ (0 as control, 0.2, 0.5 mM, 0.5 mM pre-treated with MnTABAP) and labeled with MTDR (red) for the identification of mitochondria and Hoechst33342 (blue) to identify the nucleus. C3aR were identified using Alexa-488 labeled secondary antibodies (green). **(B)** Quantitative analysis of C3aR relative fluorescence intensity on the entire cell (left graph), and normalized number of C3aR particles colocalized with MTDR fluorescence (identified as yellow particles) (right graph). **(C)** Western blot analysis for C3aR was performed in total cellular homogenates (C3aR and β-actin loading control) and mitochondrial fractions (mtC3aR and mitochondrial Cox IV protein loading control) of control and H_2_O_2_-treated ARPE-19 cells. **(D)** C3aR levels in total cellular homogenates (left graph) did not change with treatment; wherease mtC3aR were elevated with H_2_O_2_-treatment in a dose-dependent manner, an effect that could be reversed by pre-treatment with MnTBAP prior to the addtion of H_2_O_2_ (right graph). **(E)** Orthogonal projections of C3aR colocalization on MTDR in 0.5 mM H_2_O_2_ treated ARPE 19 cells and corresponding fluorescence intensity profile measurements of 3 different colocalized particles (#1–3). **(F)** Immunolabeling of C3aR on MTDR-labeled mitochondria isolated from control (left panel) and 0.5 mM H_2_O_2_-treated cells (middle), using no anti-C3aR antibody as a control (right), and its quantification. **(G)** HeLa cells are used as a positive control for C3aR receptor expression. Cells were stained with the antibody against C3aR (green) and colabeled with wheat germ agglutinin (WGA, red) to identify the plasma membrane, TMRM (magenta) for the identification of mitochondria and Hoechst 33,342 (blue) for the nucleus. Positive labeling was identified on the plasma membrane (left) and mitochondria (right); the lack of signal in the absence of the primary antibody documents the lack of non-specific binding of the secondary antibody (middle). **(H)** Hek293 cells are used as a negative control for C3aR receptor expression. Lack of C3aR was confirmed by immunolabeling on Hek293 cells (left) and western blot analysis on mitochondrial fractions (mtC3aR and mitochondrial Cox IV protein loading control), using control (left lane) and H_2_O_2_-treated ARPE-19 cells (middle lane) for comparison.Scale bar in **(A)** 10 μm (top row) and 5 μm (bottom row). *n* = 3 individual imaging experiments, multiple cells each, one-way ANOVA, *T*-test: *P < 0.05, **P < 0.001. *n* = 3 samples for Western blotting, Kruskall–Wallis test, Mann–Whitney *U*-test: *P < 0.05.

Previouosly we have demonstrated the presence of functional plasma membrane C3aR in C3a-stimulated ARPE-19 cells by ratiometric calcium imaging and immunohistochemistry in subconfluent cells (23). Here we examined C3aR distribution in fully differentiated monolayers using confocal microscopy and in mitochondrial isolates using Western blot analysis. First, to confirm antibody specificity, immunolabeling and Western blot analysis for C3aR was performed on HeLa cells (Figure 1G), which have been shown to constitutively express C3aR (24) and on HEK293 cells (Figure 1H), which are C3aR negative (25). The results were confirmed with a second antibody raised against C3aR in ARPE-19 and HEK293 cells using immunolabeling and Western blot analysis (Supplementary Figure 2), which provided identical results. Second, the technique of colocalization beween C3aR immunoreactive particles and MTDR labeled mitochondria was confirmed in orthogonal projections (Figure 1E), with Z-stack images and cross-correlation efficient obtained with JACoP plugin in ImageJ software (Supplementary Figure 1) as well as isolated mitochondria incubated with anti-C3aR antibodies (Figure 1F). Having validated our approach, in control cells, C3aR immunoreactivity was mostly identified on the plasma membrane, with very little present in the intracellular compartments (Figure 1A), including mitochondria and lysosomes (Supplementary Figure 4). Upon the induction of oxidative stress, overall C3aR immunofluorescene was slightly but significantly increased in a stepwise fashion when comparing 0.2 and 0.5 mM H_2_O_2_ (Figure 1A, middle panel). The induction of oxidative stress upon H_2_O_2_ exposure was validated by imaging of reactive oxygen species (ROS) and superoxide anion and hydroxyl radicals using H_2_DCFDA and CellRox Deep Red, respectively (Supplementary Figure 3), and inhibition was confirmed using an antioxidant N-Acetyl Cysteine (NAC) and the superoxide dismutase mimeric Mn(III)tetrakis (4-benzoic acid) porphyrin (MnTBAP) prior to addition of H_2_O_2_ (Supplementary Figure 3). Interestingly, after H_2_O_2_ treatment, the number of C3aR positive fluorescent particles on MTDR-positive mitochondria were increased 2–3 fold (Figure 1A middle panel and Figure 1B right panel, Supplementary Figure 4B). Pretreated with MnTBAP inhibited this incrase (Figures 1A,B). The number of particles on lysosomes stayed the same (Supplementary Figure 4B). The observations on translocation of C3aR to mitochondria were confirmed by Western blot analysis, documenting a transfer of C3aR to the mitochondrial compartment (Figure 1C). While levels for C3aR in cellular homogenates remained constant between control and H_2_O_2_ treated cells (Figure 1D, bottom left graph), when analyzing purified mitochondrial protein, C3aR levels were significantly higher in samples derived from cells that were treated with H_2_O_2_ when compared to controls (Figure 1D, bottom right graph). When mitochondrial fractions were analyzed from cells pre-treated with a superoxide dismutase mimetic MnTBAP prior to the addition of 0.5 mM H_2_O_2_, the translocation of C3aR immunoreactivity to the mitochondria was significantly reduced (Figures 1C,D, blot and bottom right graph). Likewise, pretreatment of cells with NAC inhibited the H_2_O_2_-induced rise in C3aR protein levels in isolated mitochondria (Supplementary Figure 3F).

**Figure 2:**
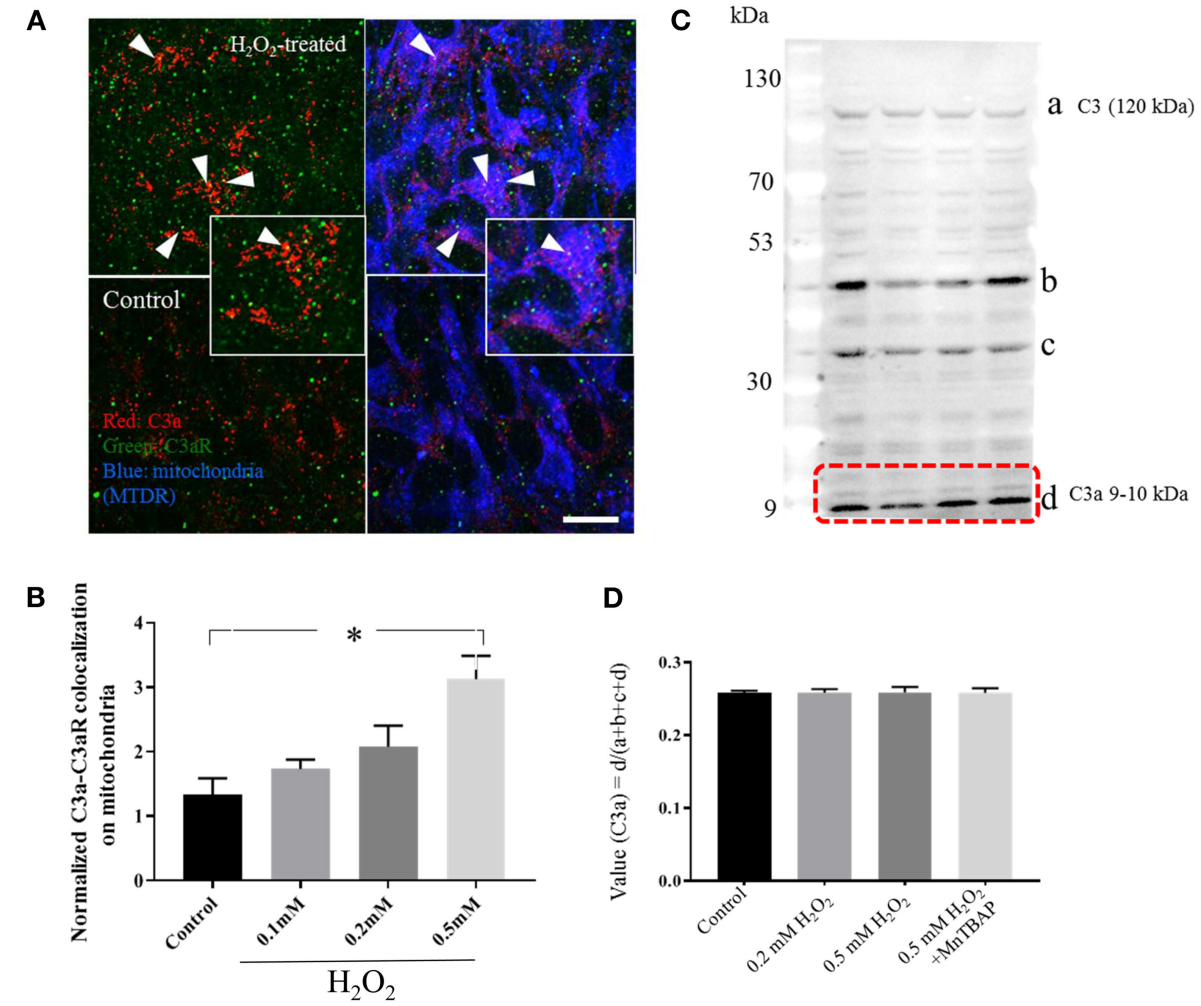
Intracellular C3a-C3aR colocalization on mitochondria. **(A)** Confocal images of C3a and C3aR immunolabeling on mitochondria in ARPE-19. C3a- (red) and C3aR-fluorescent particles (green) distribute on mitochondria identified by MTDR (false-colored blue) in control and 0.5 mM H_2_O_2_-treated ARPE-19 cells. The insets provide higher magnification images. **(B)** Quantitative analysis of C3a-C3aR colocalization (yellow) demonstrates a dose-dependent increase. **(C)** Western blot analysis for C3a immunoreative contents in the cytoplasm of control cells and those treated with H_2_O_2_, or pre-treated with MnTBAP prior to the addition of H_2_O_2_. Anti-C3a antibody recognized 4 bands [~120, ~47, ~35, and 9–10 kDa (C3a fragment)]. **(D)** Quantitative analysis, calculating the ratio of the C3a fragment over the total protein content recognized by the anti-C3a antibody. Scale bar in **(A)** 10 μm. *n* = 3 individual imaging experiments, multiple cells each, one-way ANOVA, *T*-test: *P < 0.05. *n* = 3 samples for Western blotting, Kruskall–Wallis test, Mann–Whitney *U*-test.

We have previously shown that oxidative stress induced by smoke increased C3 mRNA expression and extracellular C3a protein levels in ARPE-19 cells, but did not examine intracellular C3a (26). Here, when analyzing cytosolic C3a levels normalized to the total C3α chain cleavage products (C3α, C3α-47, C3α-37, and C3a) (Figure 2C), intracellular C3a levels were found not to be affected by H_2_O_2_ (Figure 2D). Next, we tested whether C3a binding to its receptor on mitochondria was increased upon H_2_O_2_ treatment by determining C3a-mtC3aR colocalization by immunocytochemistry (Figure 2A). C3a-mtC3aR colocalization was increased in a H_2_O_2_ concentration depended manner (Figure 2B). These results suggest that oxidative stress, rather than increasing the intracellular C3a concentration, increases the binding probability of C3a to its mtC3a receptor.

### C3aR Trafficking to Mitochondria Requires Endosomal-to-Mitochondrial Cargo Transfer

Termination of plasma membrane receptor signaling has been recognized to involve internalization by endocytosis (27), which has been confirmed for C3aR (28, 29). Receptors can then be delivered back to the plasma membrane (30), delivered for lysosomal degradation (30) or delivered via early endosomes to other organelles (31).

Our results, demonstrating a dose-dependent increase in C3aR in the mitochondria fraction in the context to stable total C3aR levels, suggest that plasma membrane C3aR is internalized upon H_2_O_2_ treatment. For this hypothesis to be true, an increase in extracellular C3a upon H_2_O_2_ stimulation and a lack of receptor internalization in the presence of a C3aR antagonist is required.

**Figure 3:**
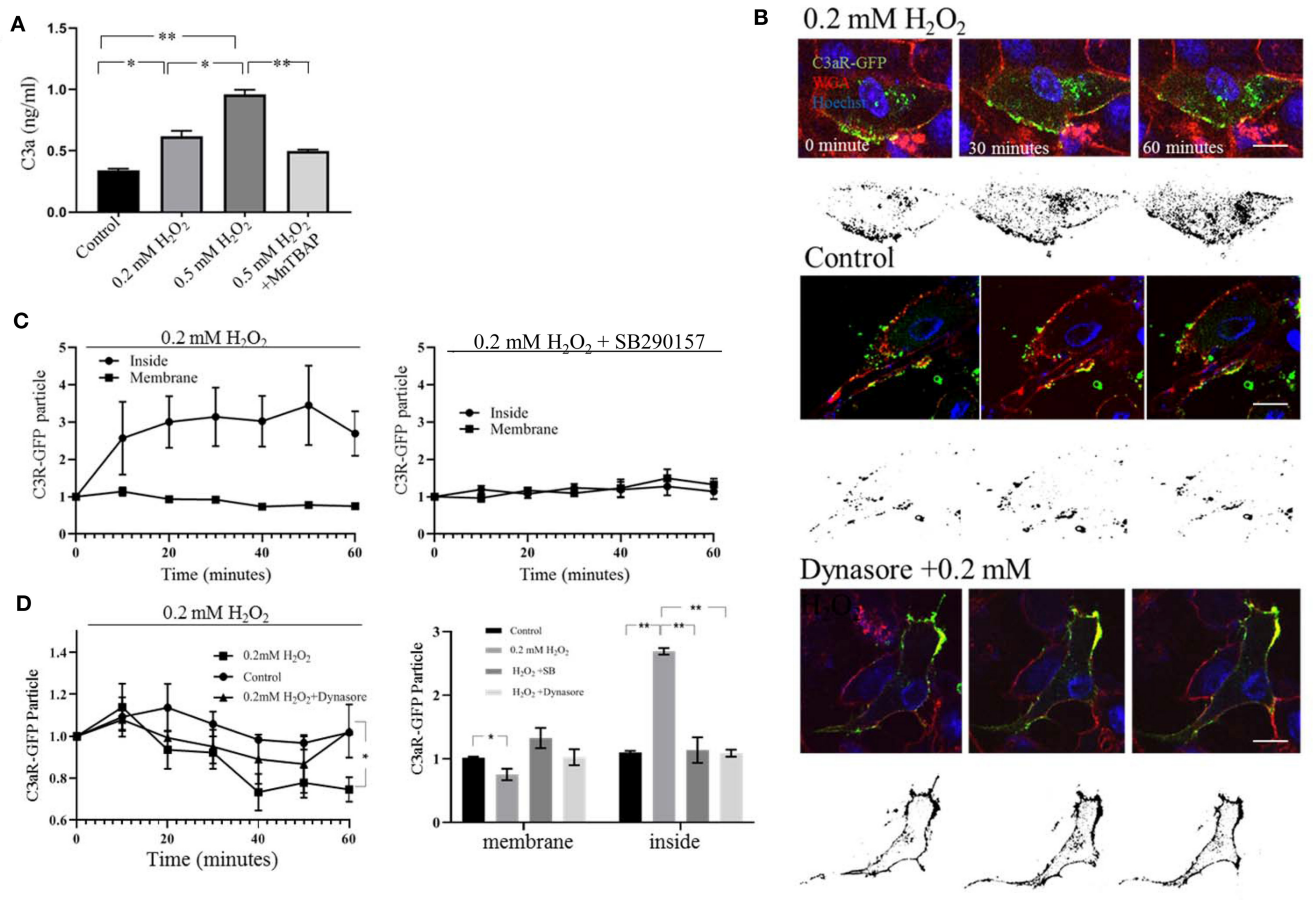
Live cell imaging of plasma membrane C3aR-GFP internalization. **(A)** ELISA assay documenting a dose-dependent increase of C3a in the culture medium 45 minutes after H_2_O_2_ treatment, an effect that could be blunted by the cell-permeable superoxide dismutase mimetic and peroxynitrite scavenger Mn-TBAP. **(B)** Representative images captured from the time lapse analysis of C3aR-GFP internalization from the ARPE-19 plasma membrane to the intracellular compartment in response to 0.2 mM H_2_O_2_ stimulation (top), no treatment (control, middle), and 0.2 mM H_2_O_2_ in the presence of the endocytosis inhibitor, dynasore (bottom). The color image represents C3aR-GFP (green), WGA (red) to label plasma membranes and Hoechst (blue) to identify nuclei. The black and white (binary) image represents in the intracellular C3aR-GFP that is no longer colocalized with the WGA signal, which enables documentation of transfer into the intracellular compartment over time. **(C)** Quantitative analysis of normalized changes of C3aR-GFP fluorecence colocalized with WGA (red) (=C3a-GFPmem), and the internalized C3aR-GFP particles (=C3a-GFPin) over time derived from the time lapse data. In cells treated with H_2_O_2_-treated (left graph) the C3aR-GFP population on plasma mebrane remains mostly stable, wherease the levels in the intracellular compartment increase. In cells pre-treated with SB290157 prior to the addition of H_2_O_2_, no translocation was observed (right graph). **(D)** Quantitiative analysis of C3aR-GFP particles on the plasma membrane over time (left graph) and at the 60 minute time point, comparing membrane and intracellular localization (right graph). In cells treated with H_2_O_2_, membrane localization of C3aR-GFP is decreased, wherease levels in the intracellular compartment increase. In cells pre-treated with the endocytosis inhibitor dynasore or SB290157 prior to the addition of H_2_O_2_, no translocation was observed. Scale bar in (A) 10 μm. *n*=3. One-way ANOVA, *T*-test: *P < 0.05, **P < 0.001.

First, C3a ELISA measurements revealed that H_2_O_2_ treatment increased extracellular C3a in a dose-dependent manner, an effect that could be inhibited by MnTBAP (Figure 3A). Second, this hypothesis was tested by live-cell imaging in cells transfected with C3aR-GFP (Figure 3B). In control cells, C3aR-GFP particles were localized to the plasma membrane identified by WGA (Figure 3B, middle panel). The addition of H_2_O_2_ shifted the number of C3aR-GFP particles from the plasma membrane (Figure 3B, top panel) to the intracellular compartment (Figure 3C, left graph), such that C3aR-GFP colocalization with WGA was reduced by H_2_O_2_ treatment, and C3aR-GFP distribution in the intracellular compartment was increased (Figure 3C), identifying a significant negative correlation between membrane bound and intracellular C3aR-GFP (*r* = −0.48). Internalization required C3aR activation, as inhibition of C3aR signaling with SB290157 prevented its internalization, resulting in no significacnt changes between the inside and membrane pools of C3aR-GFP (*r* = 0.58) (Figure 3C, right graph; Figure 3D, bar graph).

Endocytosis, whereby which vesicles buds into the cell, requires the GTPase dynamin. Pre-treatment with the dynamin inhibitor dynasore prevented the translocation of C3aR-GFP into the intracellular space as documented by imaging (Figure 3B). At 60 min the reduction of C3a-GFP on the membrane was prevented (Figure 3D, left graph), as was the increase in C3aR-GFP distribution in the intracellular compartment (Figure 3D, bar graph).

Delivery of proteins to mitochondria occurs most likely via early endosomes through membrane contact sites (31). A marker suitable for characterizing transport from early to late endosomes and from late endosomes to lysosomes is Rab7 (32); a marker unique for lysosomes is Lamp1 (33). ARPE-19 cells were cotransfected with C3aR-GFP/Rab7-RFP or C3aR-GFP/Lamp1-RFP, and mitochondria identified with MTDR. Since there is overlap in organelles labeled with Rab7 and Lamp1 (both label lysosomes), signals were characterized by imaging. Signals binned based on size of the labeled organelles identifyed 2 non-overlapping populations, small organelles (0.17 ± 0.61 μm^2^) characterized by Rab7-GFP labeling only, large ones (3.17 ± 0.23 μm^2^) labeled by Lamp1, and an intermediate sized one that is labeled by both Rab7 and Lamp1. This suggests that the small organelles labeled by Rab7-RFP represent endosomes (Supplementary Figure 5), the larger Rab7 positive organelles represent lysosomes. A cutoff for organelle size of 2.05 μm^2^ was chosen to represent early endosomes, representing 95% of the Rab7-RFP, but only 5% of the Lamp1-RFP signal (Supplementary Figure 5).

**Figure 4:**
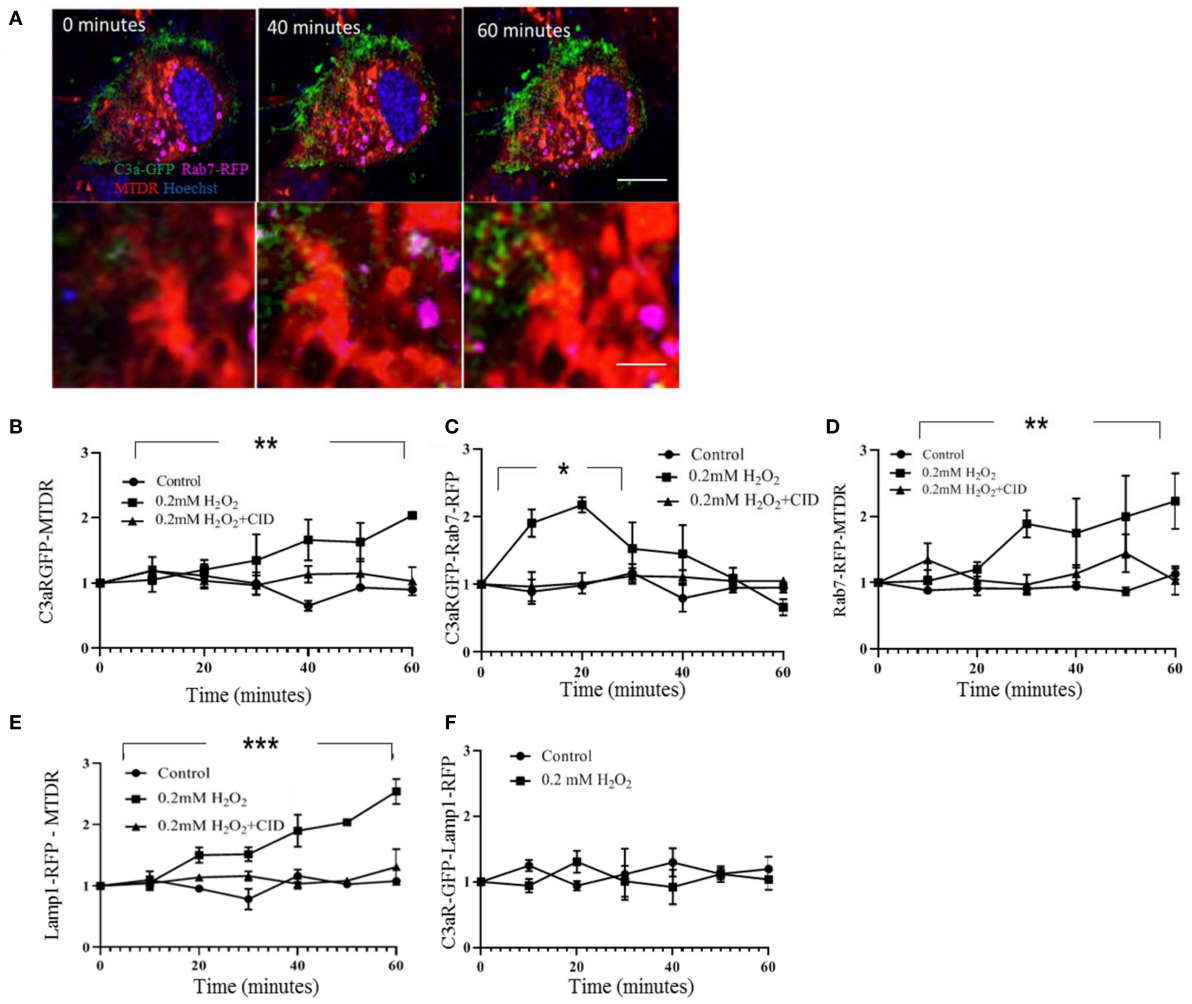
Live cell imaging analysis documenting C3aR-GFP trafficking to mitochondria. ARPE-19 cells were transiently transfected with C3aR-GFP (green) and the endosome marker Rab7-RFP (false colored magenta). For imaging, mitochondria were labeled with MTDR (red) and nuclei were identified with Hoechst (blue). **(A)** Representative time lapse images of 0.2 mM H_2_O_2_-treated cells are indicated, allowing for analysis of C3aR-GFP and MTDR as well as C3aR-GFP and Rab7-RFP colocalization. **(B–F)** Quantitative analysis of colocalization of respective markers over time, documenting the transfer of C3aR-GFP to mitochondrial via endosomes. **(B)** C3aR-GFP colocalization with MTDR increased over time in H_2_O_2_ but not control cells; an effect that could be inhibited by CID1067700. **(C)** C3aR-GFP colocalization with Rab7-RFP-positive endosomes (<2.05 μm^2^, see Supplementary Figure 5 for rationale) increased transiently in H_2_O_2_ but not control cells; an effect that could be inhibited by CID1067700. **(D)** Finally, Rab7-RFP and MTDR colocalization increased over time in H_2_O_2_ but not control cells; an effect that could be inhibited by CID1067700. Together the data in B-D show the transient localization of C3aR to endosomes, the increase in contact between mitochondria and endosomes and the accumulation of C3aR in mitochondria over time, an effect that is dependent on Rab7 signaling. **(E)** In cells transiently transfected with C3aR-GFP and the lysosome marker Lamp1-RFP (Supplementary Figure 6), contacts between lysosomes and mitochondria increased over time in H_2_O_2_ but not control cells; an effect that could be inhibited by CID1067700. **(F)** However, colocalization between lysosomes and C3aR-GFP was not affected by H_2_O_2_ exposure. Scale bar in **(A)** 10 μm. *n* = 3 individual imaging experiments, multiple cells each, one-way ANOVA, *T*-test: *P < 0.05, **P < 0.001, ***P < 0.0001.

Transport of C3aR-GFP from the plasma membrane to the mitochondria via endosomes upon H_2_O_2_ stimulation was analyzed in multiple steps (Figure 4A). First, C3aR-GFP colocalization with MTDR increased ~2-fold upon adding H_2_O_2_ by 60 min (Figure 4B). Second, colocalization between C3aR-GFP and the Rab7-RFP particles increased transiently by ~2-fold, peaking at ~20 min after the addition of H_2_O_2_ and returning to baseline (Figure 4C). This peak in colocalization could be inhibited by a competitive inhibitor of nucleotide binding by Ras-related GTPases, CID1067700 (34) (Figure 4C). And third, Rab7-RFP colocalization with mitochondria increased ~2-fold upon addition of H_2_O_2_ when compared to control, starting within 20–30 min; which could be inhibited by CID1067700 (Figure 4D). In contrast, while Lamp1-RFP labeled lysosomal contacts with mitochondria are higher after H_2_O_2_ treatment (Figure 4E, Supplementary Figure 6), there is no change in the number of C3aR-GFP and Lamp1-RFP colocalized particles (Figure 4F, Supplementary Figures 6, 7). Overall, this data suggests that under oxidative stress and in the presence of C3a, C3aR-GFP is removed from the plasma membrane by endocytosis (dynasore dependent), and transferred, in part, to mitochondria in an endosome (identified by Rab7) manner.

### mtC3aR Activation Enhanced Mitochondrial Ca^2+^ Uptake

**Figure 5:**
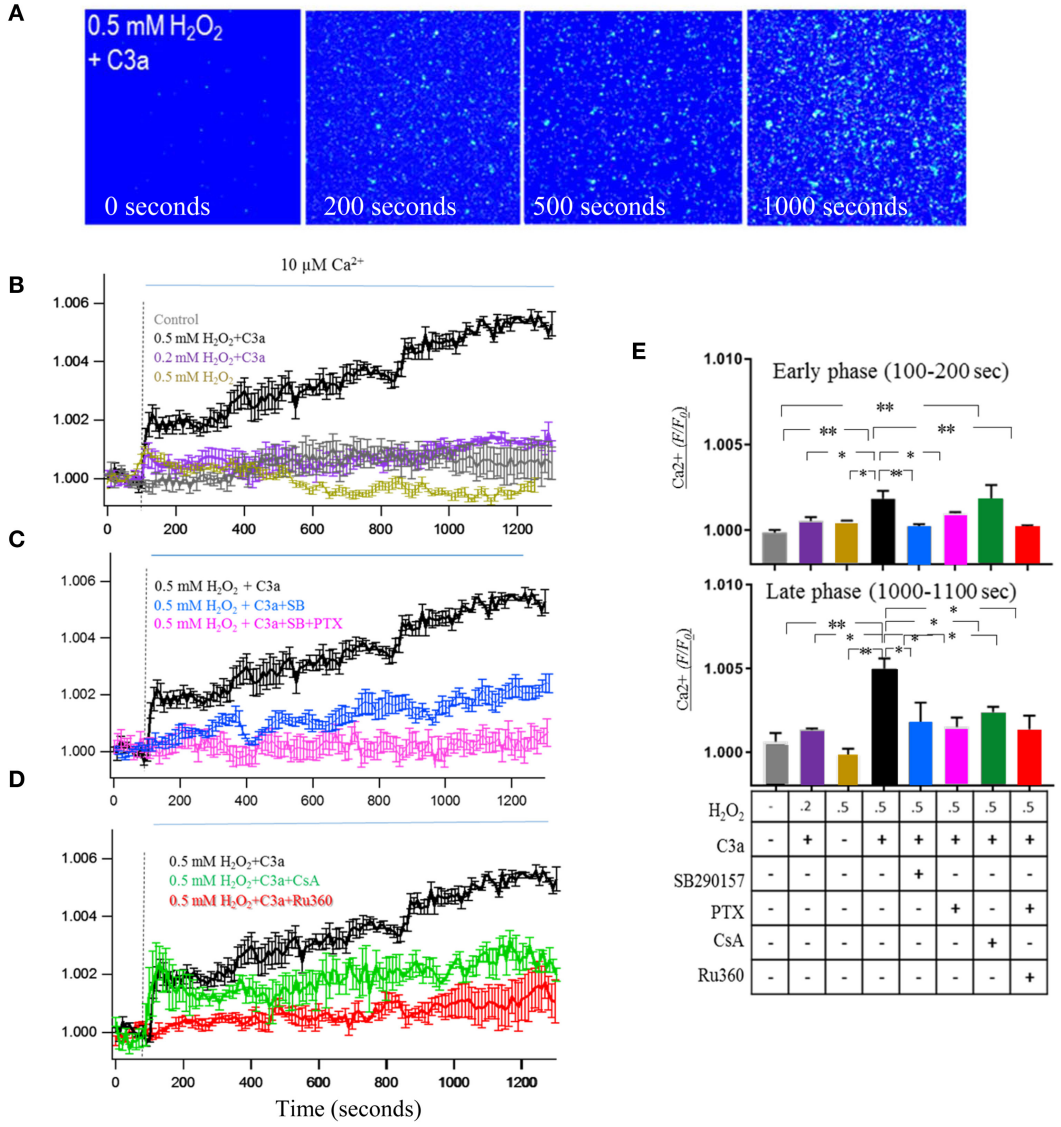
mtC3aR activation enhances mitochondrial Ca^2+^ uptake. MTDR and Fluo-8 AM pre-labeled mitochondria were isolated under caclium free conditions to enable calcium imaging in MTDR-positive organelles. Mitochondria were isolated from control cells and cells exposed to 0.2 and 0.5 mM H_2_O_2_ to enable mitochondrial transfer of C3aR. In all experiments, C3aR activation was triggered by 260 nM C3a, unless otherwise noted. The Fluo-8 AM signal was analyzed at 2 time points after the addition of calcium, the early [100–200 s, **(E)** top graph] and late phase [1,000–1,100 s, **(E)** bottom graph]. **(A)** A representative color image of calcium flux measured with Fluo-8 AM on MTDR-labeled isolated mitochondria from 0.5 mM H_2_O_2_-treated cells which time point?. C3aR activation was mediated by 260 nM C3a, calcium uptake was triggered by exposure to 10 μM Ca^2+^. **(B)** Fluo-8 AM fluorescence intensity changes measured over time in mitochondria isolated from no-treatment (control, gray), 0.2 mM H_2_O_2_-treated (purple) and 0.5 mM H_2_O_2_-treated (black and yellow) ARPE-19 cell. In the absence of C3a, calcium exposure triggered a slow wave in control mitochondria, compared to a fast wave in 0.5 mM H_2_O_2_ mitochondria. In the presence of C3a, calcium exposure triggered an early and late calcium response in 0.5 mM H_2_O_2_ mitochondria, but only the early response in 0.2 mM H_2_O_2_ mitochondria. **(C)** Both the early and late phase of the calcium response triggered in H_2_O_2_ mitochondria triggered by C3a and Ca^2+^ could be inhibited by the C3aR antagonist SB290157 (blue) and the Gαi G-protein inhibitor PTX (pink). **(D)** Fluo-8 AM fluorescence intensity changes measured over time in the presence of inhibitors known to block the mitochondrial calcium uniporter (mCU, Ru360) and the mitochondrial permeability transition pore (mPTP, cyclosporin A, CsA). Ru360 blocked the Ca^2+^-induced calcium uptake, CsA had no effect on the rapid Ca^2+^ elevation, but blocked the late phase of the response. **(E)** The normalized Fluo-8 AM intensity changes of the early phase (100–200 s) and late phase (1,000–1,100 s) are analyzed and presented for comparison. Scale bar in **(A)** 10 μm. *n* = 3 individual imaging experiments, multiple cells each, one-way ANOVA, *T*-test: *P < 0.05, **P < 0.001.

One of the key functions of mitochondria is handling of Ca^2+^ (35), inspiring the question whether mtC3aR activation might alter Ca^2+^ uptake in isolated mitochondria. For this experiment, mitochondria in intact cells were labeled with MTDR and Fluo-8 AM prior to the isolation procedure to enable their future identification and ability to image Ca^2+^; followed by isolation of mitochondria from cells in the presence of EDTA and BAPTA-AM to prevent loading of cytosolic Ca^2+^ into mitochondria (18). C3aR activation was triggered by the addition of 260 nM C3a (36), followed by the addition of 10 μM of CaCl_2_ solution to trigger Ca^2+^ uptake (stippled line in Figures 5B–D). Ca^2+^ fluorescence was quantifyed for the early (100–200 s) and late (1,000–1,100 s) component of the response (Figure 5E). Upon addition of 10 μM Ca^2+^, Fluo-8 AM fluorescence in control mitochondria showed a slow increase in signal, peaking at 600–800 s, and then decreasing back to baseline by 1,200 s (Figures 5B,E). In contrast, mtC3aR activation in mitochondria isolated from cells treated with 0.5 mM H_2_O_2_ and stimulated with C3a showed a rapid and large increase in fluorescence within 30 s after adding Ca^2+^, followed by a slow and continuous elevation that did not plateau prior to the end of the measurements (Figures 5B,E). This is in contrast to the mitochondria isolated from cells treated with 0.5 mM H_2_O_2_ that were not stimlated with C3a, in which only the early, but not the late phase of Ca^2+^ uptake could be triggered (Figures 5B,E). Finally, mitochondria isolated from cells treated with 0.2 mM H_2_O_2_ showed an intermediate phenotype, including the early increase in fluorescence amplitude upon the addition of Ca^2+^ and C3a, but lacking the continuous rise in signal (Figures 5B,E).

The pharmacological underpinnings of this Ca^2+^ response was followed up in mitochondria isolated from cells treated with 0.5 mM H_2_O_2_. First, the influx of Ca^2+^ could be inhibited with SB290157 (Figures 5C,E) or PTX (Figures 5C,E). Second, the contributions of the mitochondrial calcium uniporter (mCU) and mitochondrial permeability transition pore (mPTP) to the Ca^2+^ response were investigated. Physiological levels of Ca^2+^ are known to activate the mCU whereas the mPTP plays a role in mitochondrial Ca^2+^ homeostasis under pathological conditions (37). In addition, the dependence of mPTP gating on mCU activity has been demonstrated (38). The addition of the mitochondrial Ca^2+^ uniporter blocker Ru360 blocked the Ca^2+^-induced calcium uptake (Figures 5D,E). Cyclosporine A (CsA), a mitochondrial transition pore inhibitor, did not show any effect on the rapid Ca^2+^ elevation, but it inhibited the continuous elevation during the late phase (Figures 5D,E). These experiments demonstrated that the early phase of the Ca^2+^ response is driven by Ca^2+^ influx through mCU, the late phase by the mPTP, and that activation of the mCU was required to trigger influx of Ca^2+^ through the mPTP.

### Effects mtC3aR Activation on Mitochondrial Respiration

**Figure 6:**
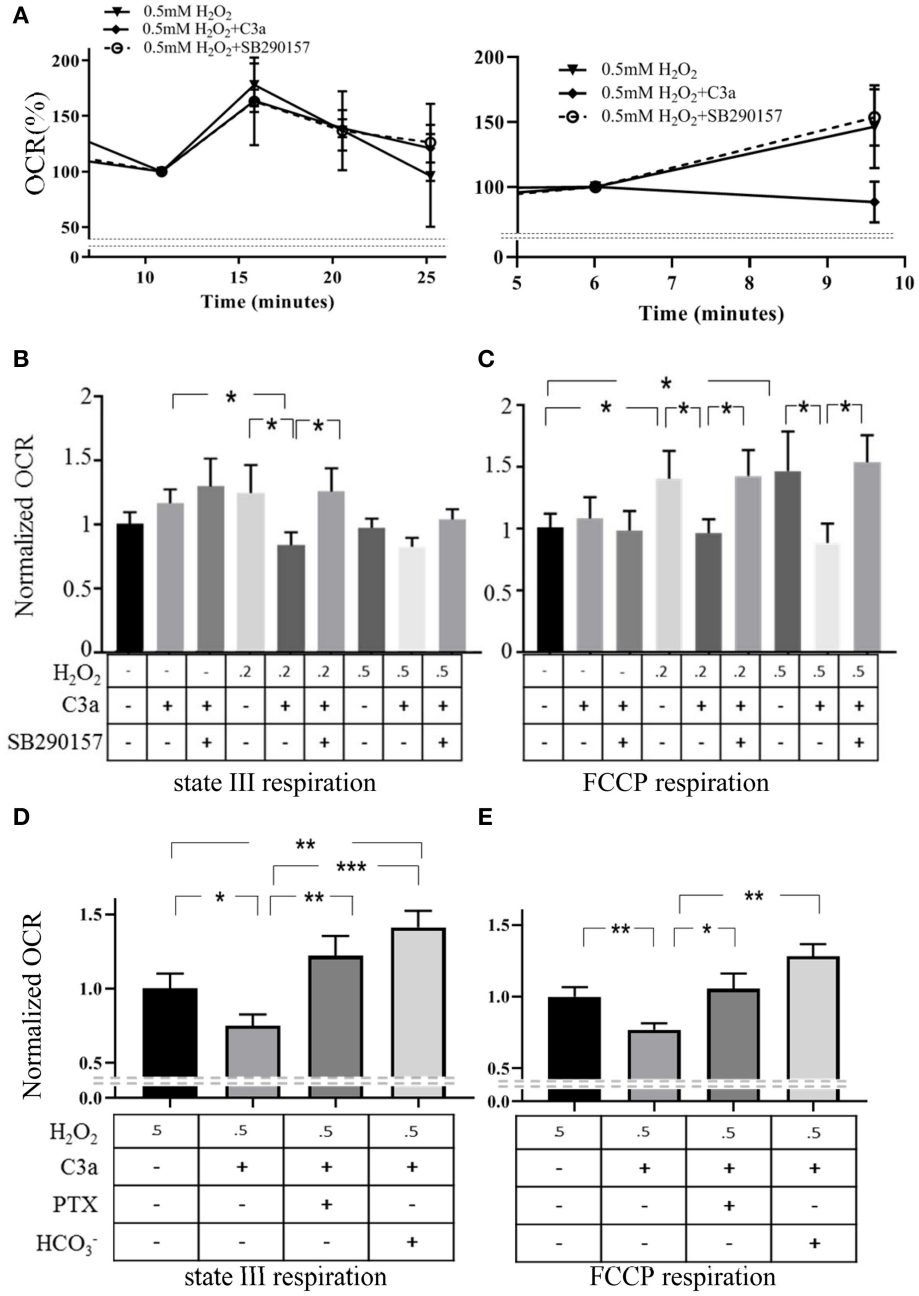
mtC3aR activation inhibited mitochondrial electron transport chain activiy. High resolution respirometric XFe96 Seahorse assays were utilized on mitochondria isolated from control cells and cells exposed to 0.2 and 0.5 mM H_2_O_2_ to drive C3aR to the mitochondria. In all experiments, C3aR activation was triggered by 260 nM 30 min prior to the assay, unless otherwise noted. Oxygen consumption rates (OCR) are recorded every 5 min to determine ADP driven state III respiration and the maximal respiratory capacity. Those 2 parameters were analyzed upon injection of ADP (4 mM) and the protonophore FCCP (6 μM), respectively. All data was normalized to the mean OCR value of the control at peak state III or FCCP response. **(A)** Examples of state III respiration (left) and FCCP response (right) are presented from mitochondria isolated from H_2_O_2_-treated ARPE-19 and treated with C3a and C3a + C3a antagonist, respectively. Quantitative OCR results of **(B)** state III respiration and the **(C)** FCCP response of isolated mitochondria (control, 0.2, 0.5 mM H_2_O_2_) stimulated with C3a and/or C3a and C3aR antagonist SB290157 are presented. C3a inhibited ADP drive (state III respiration) and FCCP response in stressed mitochondria, an effect that could be reversed by C3aR antagonism. To examine the involvement of G-protein signaling and the known downstream signaling molecule of Gαi in mitochondria, the soluble adenylyl cyclase, **(D)** state III respiration and the **(E)** FCCP response of isolated mitochondria (0.5 mM H_2_O_2_) stimulated with C3a was determined. Normalized OCR results demonstrated that the Gαi inhibitor PTX and the soluble adenylyl cyclase activator ${\text{HCO}}_{3}^{-}$ reversed the inhibtory effect of C3a on ADP drive as well as on maximal respiratory capacity. Data from 9 to 25 wells in 3–5 independent experiments are reported. One-way ANOVA, *T*-test: *P < 0.05, **P < 0.001, ***P < 0.0001.

A second key role of mitochondria is the generation of ATP through activation of the electron transport chain. Next we tested mtC3aR activation effects on mitochondrial repiratory function in isolated mitochondria, comparing mitochondria isolated from control cells and cells treated with H_2_O_2_ to increase mtC3aR translocation. High-resolution respirometric assays were performed under optimal Ca^2+^ conditions for ARPE-19 cell mitochondria (3 nM) previously identified (18). Two aspects of mitochondrial function were examined, the increase in respiration rate in response to ADP (ADP drive or state III respiration) and the maximum respiratory rate (FCCP response) (Figure 6A).

Respiration rates (oxygen consumption rate, OCR) in response to ADP in mitochondria isolated from control cells were identical irrespective of whether they were exposed to vehicle or 260 nM C3a (Figures 6A,B). C3aR activation, however, significantly inhibited OCR in mitochondria from H_2_O_2_ treated cells (Figures 6A,B), and co-administration of C3a and SB290157 reversed the inhibition (Figures 6A,B).

Uncoupled respiration was assessed upon addition of the protonophore FCCP (Figures 6A,C). OCR in response to FCCP was increased in mitochondria from cells treated with H_2_O_2_ when compared to those isolated from control cells. Activation of mtC3aR inhibited the FCCP response in mitochondria from H_2_O_2_-treated cells (Figures 6A,C), and the C3aR antagonist reversed the C3a-mediated inhibition (Figures 6A,C). Mitochondria isolated from control cells did not show any change by C3aR activation and antagonist treatment.

mtC3aR signaling was further assessed based on the downstream signaling components characterized in part by Ca^2+^ imaging (Figure 5). C3aR is associated with Gαi that inhibits a soluble form of adenylyl cyclase (sAC), which we have shown to be involved in control of mitochondrial respiration (18). Respiration assays were repeated in mitochondria isolated from 0.5 mM H_2_O_2_-treated cells in the presence of PTX and the sAC activator ${\text{HCO}}_{3}^{-}$ (Figures 6D,E). The reduction in ADP drive induced by mtC3aR activation could be reversed by PTX or ${\text{HCO}}_{3}^{-}$ (Figure 6D). Likewise, both PTX and ${\text{HCO}}_{3}^{-}$ reversed the reduction in FCCP response induced by mtC3aR activation (Figure 6E).

### mtC3aR Expression on Trans-Mitochondrial Cybrids Cell From AMD Patient

The results presented thus far suggest that transiently induced oxidative stress by H_2_O_2_ results in the transfer of C3aR to mitochondria where C3a activation increases pathological Ca^2+^ influx and reduces ATP production. Here we extend this set of experiments, investigating mtC3aR localization and mitochondrial function in trans-mitochondrial ARPE-19 cybrids (39), in which the single nucleotide polymorphisms SNP G13708A-1210bp (J-haplogroup) and SNP C7028T-308bp (H-haplogroup) lead to differences in ATP production and different disease risk (15, 40–42).

**Figure 7:**
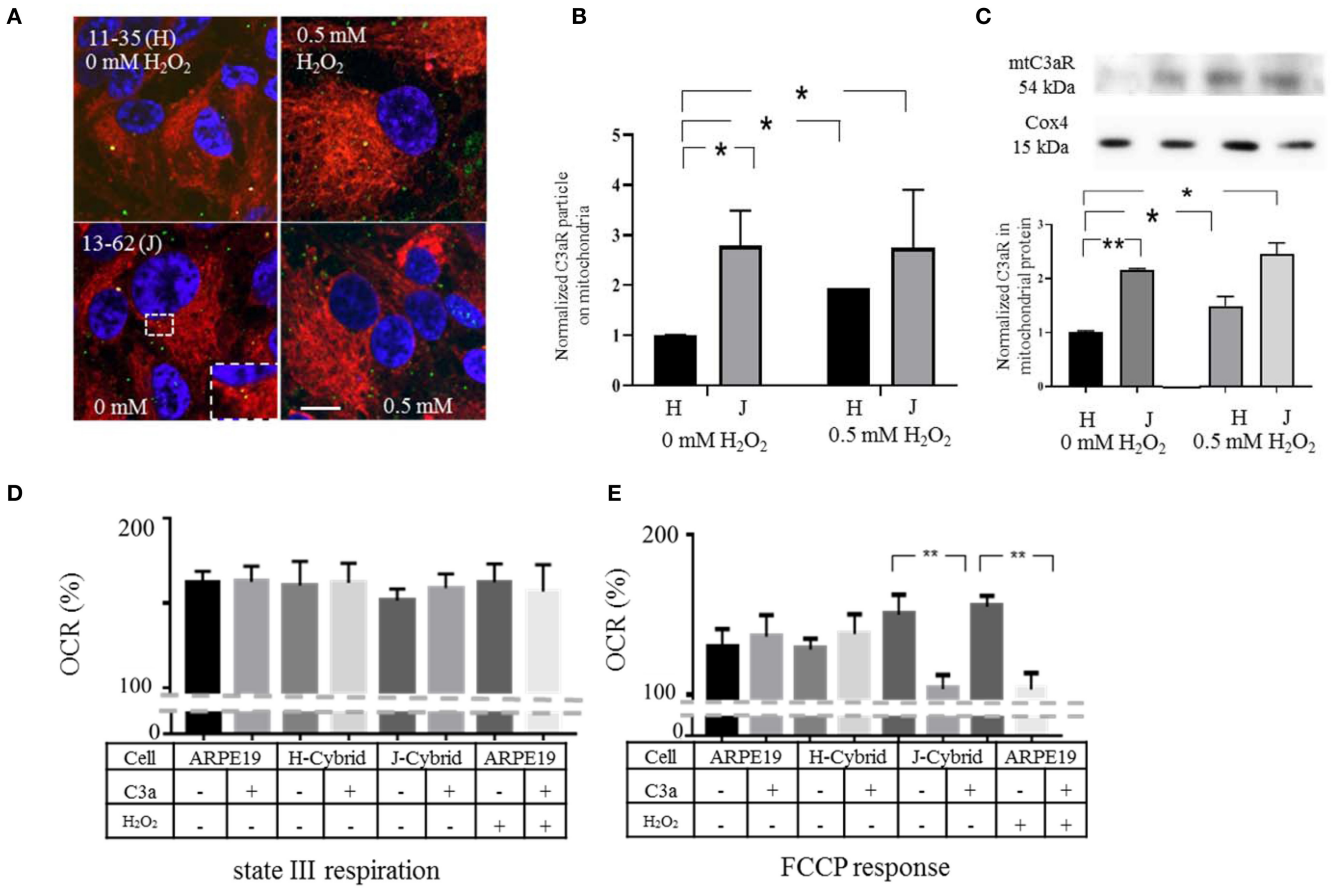
ARPE-19 cell cybrids that confer disease risk exhibit high levels of mtC3aR and their activation ATP synthesis. Transmitochondrial cybrid ARPE-19 cells contain identical nuclear but different mitochondrial DNA. The J-haplotype is associated with reduced metabolism and increased disease risk when compared to the H-haplotype. **(A)** Confocal microscopy was used to analyze the distribution of C3aR by immunohistochemistry (green). Mitochondria were identified by MTDR (red), the nucleus by Hoechst 33,342 (blue). Images in cells exposed to vehicle (left column) and H_2_O_2_ (right hand column) are provided. The inset highlights the mitochondrial localization (yellow). **(B)** Quantitative analysis of C3aR particle counts on mitochondria normalized to that of identified in H-cybrids exposed to vehicle. mtC3aR levels in vehicle treated J-cybrids are 3-fold higher than those of H-cybrids. And while mtC3aR levels could be increased upon 0.5 mM H_2_O_2_ exposure in H-cybrids, levels in J-cybrids could not be elevated any further. **(C)** Western Blot analysis of C3aR in mitochondrial protein fractions isolated from J- and H-cybrids confirmed the results of the imaging analysis. **(D,E)** Mitochondrial respiration was analyzed in isolated mitochondria harvested from J- and H-cybrids as well as control and H_2_O_2_ treated ARPE-19 cell. XFe96 Seahorse assays were utilized as in Figure 6, determining OCR levels for state III respiration **(D)** and maximal respiratory capacity [FCCP response, **(E)]**. All values are reported as OCR %, representing the measured value normalized to its own baseline. mtC3aR were activated by the addition of C3a prior to the respirometric analysis. The FCCP response in mitochondria from J-cybrids and H_2_O_2_ treated ARPE-19 cells was susceptible to C3a inhibition, whereas those from control and H-cybrids were not. State III respiration was not affected by any of the parameters. Scale bar in (A) 10 μm. *n* = 3 individual imaging experiments, multiple cells each, one-way ANOVA, *T*-test. *n* = 3 samples for Western blotting, Kruskall–Wallis test, Mann–Whitney *U*-test. Data for Seahorse from 8 wells, one-way ANOVA, *T*-test: *P < 0.05, **P < 0.001.

Surprisingly, C3aR localization on mitochondria was 3-fold higher in J-cybrids when compared to H-cybrids in the absence of oxidative stress. In the presence of 0.5 mM H_2_O_2_ levels of mtC3aR localization could be increased in H-cybrids, whereas levels in J-cybrids could not be further increased (Figures 7A,B). These results were confirmed in mitochondrial isolates from H- and J-cybrids with and whithout H_2_O_2_ stimulation (Figure 7C).

C3aR localization on mitochondria suggest that mitochondrial respiration should be susceptible to C3a activation in J- but not H-cybrids in the absence of oxidative stress. State III respiration and FCCP responses were compared in response to mtC3aR activation in isolated mitochondria from J- and H-cybrids and compared with mitochondria isolated from 0.5 mM H_2_O_2_-treated ARPE-19 cells. OCR in response to FCCP was higher in mitochondria from cells treated with 0.5 mM H_2_O_2_ and in mitochondria isolated from J- but not H-cybrids (Figure 7E). mtC3aR acitvation on mitochondria isolated from J-cybrids inibited the FCCP response, similar to that in mitochondria from cells treated with 0.5 mM H_2_O_2_, whereas mitochondria from H-cybrids were unresponsive (Figure 7E). ADP drive could not be changed by any of the parameters (Figure 7D).

## Discussion

Here we set out to further contribute to the analysis of extra- vs. intra-cellular complement signaling in epithelial cells. Our key findings are as follows: We report for the first time that C3aR localizes to mitochondria in oxidatively stressed, H_2_O_2_-treated ARPE-19 cells. Colocalization of C3a and C3aR on mitochondria was found to increase in a dose-dependent manner. H_2_O_2_ resulted in the generation of extracellular C3a, activating and promoting plasma membrane C3aR internalization by dynamin-dependent endocytosis. C3aR is targeted via endosomal transport to mitochondria. mtC3aR-mediated G-protein (Gαi) signaling triggered mitochondrial calcium uptake through the mCU, which can trigger additional Ca^2+^ uptake through mPTP opening. Furthermore, mtC3aR activation affected respiratory function of mitochondria isolated from oxidatively stressed cells, inhibiting ADP-drive respiration and maximal respiratory capacity. Finally, in trans-mitochondrial cybrid cells with haplogroups known to alter ATP output and thus potentially generating metabolic stress, mtC3aR localization levels were significantly different. mtC3aR levels were elevated in J-cybrids that are associated with higher risk for multiple diseases, when compared to H-cybrids. Concomitantly, in respirometry assays, mtC3aR activation on isolated mitochondria decreased FCCP uncoupled responses in J- but not H-cybrids. In summary, the data suggests that both transient and long-term stress results in an increase in C3aR on mitochondria where receptor signaling contributes to pathological calcium influx and reduced mitochondrial ATP synthesis.

### Intracellular Complement

Location specific production of complement components in different tissues and cells has long been recognized. However, most recently, the observation that the complement system might not only be operative extracellularly but also intracellularly has gained traction (43). Here we find evidence of the presence of the C3a receptor and its ligand C3a intracellularly, with a significant localization to mitochondria.

C3aR is thought to be mainly distributed on the plasma membrane; however, in lymphoid cells C3aR has been identified on lysosomes (8). Previously, we showed by immunolabeling for C3aR in ARPE-19 cells that the immunofluorescent material seemed to be distributed not only on the plasma membrane but potentially also inside the cell; however, that possibility was not further investigated (36). Here, we examined intracellular C3aR localization with different methods to confirm our observation of mitochondrial distribution. C3aR was identified in intact cells by immunocytochemistry and by imaging in cells transiently expressing C3aR-GFP. Second, localization to mitochondria was confirmed in isolated mitochondria by Western blotting and confocal microscopy. And third, localization of C3aR to mitochondria was confirmed functionally by calcium imaging and respirometry assays, stimulating the receptor with C3a, and inhibiting the activity with a specific C3aR antagonist.

C3a specific immunoreactivity was identified as the typical 9 kDa band by Western blotting. By immunofluorescence, we focused on the colocalization between C3a and its receptor on mitochondria, as the α-C3a antibody is not expected to distinguish between C3a and full-length C3. Levels of C3a-mtC3aR colocalization on mitochondria increased in stressed cells, overall suggesting that intracellular C3a activates mtC3aR under stress conditions. However, these experiments do not provide evidence as to the origin of C3a. Elvington et al. have shown that subconfluent ARPE-19 cells do not express appreciable amounts of C3, but rather the conformationally altered form of C3, C3H_2_O, is taken up by endocytosis (44). Uptake was characterized by the authors as a specific mechanism of loading, and cells could subsequently release C3H_2_O. While the authors further confirmed C3a production derived from C3H_2_O in cells, that particular experiment was performed in lymphoid rather than ARPE-19 cells (44). Denny and Johnson demonstrated that cultured human endothelial cells take up ^125^I-labeled C3a, and that the labeled material is present within the cell cytoplasm (45). We have shown that ARPE-19 cells grown in monolayers express and secrete C3 from the apical and basal membranes, and that generation of C3a could be documented in the supernatant of cells stressed by smoke, a process that could be inhibited by alternative pathway inhibition (26). These results are similar to the ones presented here, that ARPE-19 cells grown in monolayers generate C3a in the extracellular milieu upon oxidative stress, triggered by H_2_O_2_ exposure. The intracellular C3a might stem from the extracellular pool as suggested by the ^125^I-labeled C3a uptake data, or generated intracellularly from C3. Many proteases have been shown to cleave C3, including kallikrein (46), trypsin, and some of the coagulation cascade enzymes, elastase as well as cathepsins B, G, and L [reviewed in Huber-Lang et al. (47)]. Most of these enzymes are present in ARPE-19 cells based on mRNA expression data (48). Cellular stimuli such as inflammation and oxidative stress might activate these enzymes intracellularly and/or extracellularly. Finally, some of the C3a, in particular that bound to C3aR on mitochondria after H_2_O_2_ stimulation, might represent extracellular C3a bound to the receptor that triggered internalization.

### C3a Receptor

C3a receptors are mainly localized to the plasma membrane. In RPE cells, plasma membrane C3aR activation has been shown by us to induce increases in free cytosolic Ca^2+^ and PI3-kinase/Akt activation, FoxP3 and FOXO1 phosphorylation (23). Termination of activity involves plasma membrane C3aR internalization (29, 49) by clathrin-coated pit-mediated endocytosis (50, 51).

C3a receptor expression is elevated in tissues of patients and models of their diseases. For example, C3aR mRNA levels are increased in the ischemic mouse cortex after middle brain artery occlusion, and C3aR immunostaining was identified on cortical neurons and astrocytes (52). Increased C3aR mRNA expression has also been identified in coronary arteries of human atherosclerosis patients (39).

Organellar C3aR localization, C3aR activation and intracellular C3 production has recently been shown in T-cells, documenting its presence on lysosomes (8), Specifically, cathepsin L-dependent cleavage of C3 into biologically active forms of C3a and C3b was demonstrated. Intracellular C3a was found to be required for homeostatic T-cell survival, while transfer of intracellular C3a bound to C3aR to the cell surface induced cytokine production upon T-cell stimulation. Interestingly, intracellular C3aR activation engaged the nutrient sensor mammalian target of rapamycin and affected levels of glycolysis (53).

Based on these observations in lymphoid cells, we posed 2 questions; the origin of mtC3aR, whether it be plasma membrane- or the lysosome-derived; and the effect of C3aR activation on metabolism. The first question was investigated with live cell imaging, tracking C3aR-GFP in the presence of markers for early endosomes, lysosomes and mitochondria in transfected ARPE-19 cell. H_2_O_2_-treatment promoted C3aR-GFP internalization into the intracellular compartment from the plasma membrane within 10 min (*r* = −0.487). C3aR-GFP colocalization with mitochondria (*r* = 0.481) increased linearly, with significant differences in levels between H_2_O_2_-treated cells and controls by 30 min. C3aR-GFP could be demonstrated in Rab7-RFP positive endosomes in a H_2_O_2_-treatment dependent manner, and the Rab7 inhibitor CID1067700 inhibited mitochondrial C3aR transfer. And while C3aR-GFP was also found to colocalize with Lamp1-RFP positive lysosomes, H_2_O_2_ treatment did not affect the level of colocalization between C3aR-GFP and Lamp1-RFP, despite the increase in lysosome-mitochondria contacts. These results were confirmed in additional imaging experiments, using C3aR labeling in lysotracker and mitotracker co-labeled cells, which showed that while the number of lysosomal C3aR particles remained constant with H_2_O_2_ treatment, the number of mitochondrial C3aR particles increased at the expense of the other compartments. Overall, these results suggest that plasma membrane C3aR was internalized and transported to the mitochondria via endosomal transport. The data does not support transfer of lysosomal C3aR to mitochondria, and the rapid time course of C3aR delivered to the mitochondria does not support this to be newly synthesized protein.

Interestingly, mtC3aR activation was found to trigger Ca^2+^ influx into mitochondria and inhibit mitochondrial respiration only in mitochondria isolated from stressed cells. The calcium uptake response was dependent on the amount of stress delivered to the cells. Low levels of stress (0.2 mM H_2_O_2_) triggered transient calcium uptake mediated by the mCU; whereas more extensive stress (0.5 mM H_2_O_2_) triggered a continuous response mediated by the mCU followed by the mPTP. This observation has significant consequences for cellular health, since influx of calcium through the mPTP is involved in triggering apoptosis (54). Second, C3a was found to regulate mitochondrial function. Specifically, C3a reduced ADP drive and the FFCP uncoupled response in mitochondria derived from stressed cells, but not in unstressed cells. Thus, in epithelial cells, mtC3aR signaling reduces metabolic output.

### GPCR on Mitochondria

C3aR is a GPCR; the α-subunit for C3aR is Gαi, as signaling is sensitive to PTX (55). Gαi activation in the cytoplasm leads to the inhibition of the cAMP-dependent pathways through inhibition of adenylate cyclase (56). In contrast, in the mitochondria, which are devoid of membrane bound adenylate cyclase (57), Gαi activation leads to the inhibition of a soluble adenylate cyclase, which, via protein kinase A-dependent phosphorylation of electron transport chain subunits, leads to decreased cellular respiration (9). We confirmed some of these observations here in isolated mitochondria. Specifically, we showed that the inhibition of respiratory function by C3aR activation could be reversed by both the G-protein inhibitor PTX and the sAC activator HCO3^−^.

Several additional GPCRs have been reported on mitochondria. The cannabinoid CB1 receptor was identified on mitochondria in hippocampal neurons. CB1 receptor activation inhibited synaptic activity and mitochondrial complex I component NDUF2 phosphorylation by inhibition of sAC-PKA signaling (9). Mitochondria in cardiac myocyte have been shown to contain serotonin receptors 5-HTR3 and 5-HTR4 (10). Interestingly, mitochondrial 5-HTR4 activation inhibited mitochondrial Ca^2+^ uptake, which could be reversed by adenylate cyclase inhibition (10). This is in contrast to mtC3aR activation enhancing mitochondrial Ca^2+^uptake. However, 5-HTR4 is coupled to a G protein with a Gαs subunit, and Gαs activation leads to the generation of cAMP as the second messenger. Taken together, our results support the role of G-protein signaling in modulating cAMP-PKA signaling in mitochondrial physiology, which can be altered by C3aR signaling.

### mtC3aR Activation in Mitochondrial Cybrids

Mitochondrial dysfunction modulated by C3aR activation could be demonstrated in oxidatively stressed cells after short-term exposure with H_2_O_2_. As a follow-up question, we sought to investigate whether long-term alterations in metabolic function due to mitochondrial haplogroups, would lead to similar changes. Cybrid ARPE-19 cells, which are cells with identical nuclear but different mitochondrial DNA, were utilized, focusing on H- and J-haplotypes. H- and J-mitochondria in human osteosarcoma cybrids have previously been shown to exhibit differences in coupling efficiency of the electron transport chain, with J-mitochondria producing less ATP and more heat, when compared to H-mitochondria producing more ATP and less heat (58). In addition, individuals with J-haplogroups are at higher risk for certain diseases, including Leber's hereditary optic neuropathy (40), accelerated progression to AIDS and death in HIV-infected individuals (41), and age-related macular degeneration (42). The ARPE-19 cell cybrids used here were original generated to study pathology in age-related macular degeneration. ARPE-19 J-cybrids have previously been shown to have significantly lower levels of ATP, increased lactate levels, and altered nuclear gene expression (15). Here we extended this analysis using Seahorse assays to analyze the effects of mtDNA variants on mitochondrial respiration. We documented that mtC3aR localization in J-cybrids was ~2-fold higher than that of H-cybrids at baseline, and that while 0.5 mM H_2_O_2_-treatment was able to trigger transfer of C3aR from the membrane to mitochondria in H-cybrids, mtC3aR could not be further increased in J-cybrids. Consequently, mtC3aR activation on J-cybrids at baseline showed similar responses in the FCCP uncoupling effect as oxidatively stressed ARPE-19 mitochondria. Oxidative stress and the J-haplotype represent risk factors for age-related macular degeneration. Thus, mtC3aR localization and activation appear to denote chronic abnormalities. This hypothesis is further supported by the observation that mitochondria are fragmented and damaged in the RPE of subjects with age-related macular degeneration (59). As mitochondrial fission events require mitochondrial Ca^2+^ overload (60), influx of calcium following mtC3aR activation might promote mitochondrial fission and damage.

## Summary

In summary, C3aR transfer from the plasma membrane to the mitochondria via endosomal particles was demonstrated in oxidatively stressed H_2_O_2_-treated cells. Mitochondrial C3a receptor activation produced two critical responses; enhanced Ca^2+^ uptake leading to increased inner mitochondrial Ca^2+^concentration, and a reduction of mitochondrial respiration resulting in reduced ATP production and impaired maximal respiratory capacity. In addition, we confirmed that mitochondrial cybrids generated with J-haplotype mitochondria showed elevated levels of mtC3aR and similar metabolic abnormalities as mitochondria from H_2_O_2_ treated cells. Overall, oxidative stress is associated with many different diseases, and thus complement activation on mitochondria might be a more common feature of pathology. Hence, mtC3aR activation and signaling might provide clues to novel therapeutic approaches in complement-dependent diseases.

## Data Availability Statement

The original contributions presented in the study are included in the article/Supplementary Material, further inquiries can be directed to the corresponding author/s.

## Author Contributions

MI: conceptualization, methodology, experimental data acquisition and analysis, and original draft preparation. GB: experimental data analysis and data curation. CB: supervision. BR: conceptualization, writing, original draft preparation, and supervision. All authors contributed to the article and approved the submitted version.

## Conflict of Interest

The authors declare that the research was conducted in the absence of any commercial or financial relationships that could be construed as a potential conflict of interest.
