# Supplementary figures and images for: Mitochondrial C3a Receptor Activation in Oxidatively Stressed Epithelial Cells Reduces Mitochondrial Respiration and Metabolism
Source: Front Immunol. 2021 Mar 5;12:628062. doi: 10.3389/fimmu.2021.628062 (PMC7973370; doi:10.3389/fimmu.2021.628062)

Figure S1

A

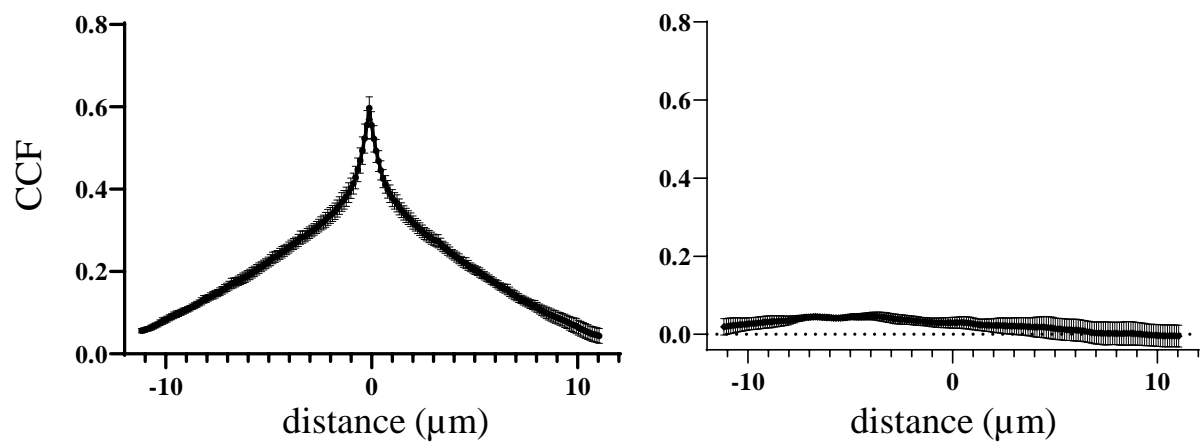

B

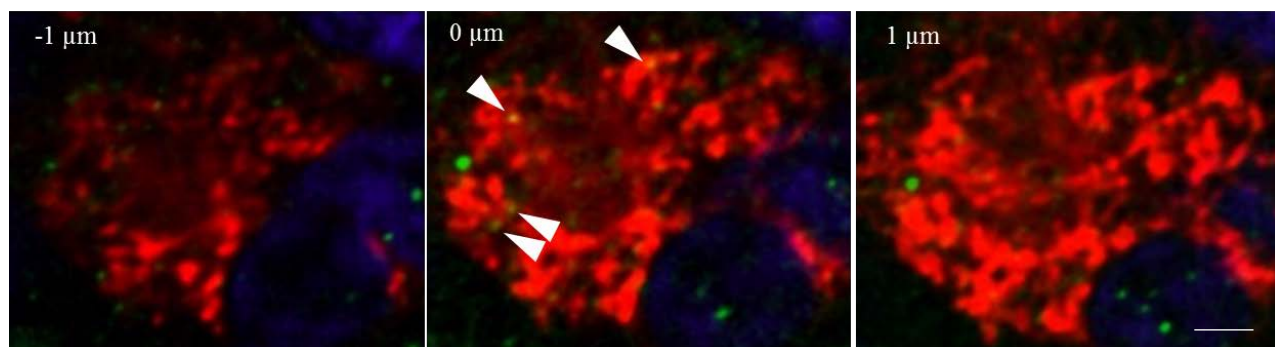

Figure S2

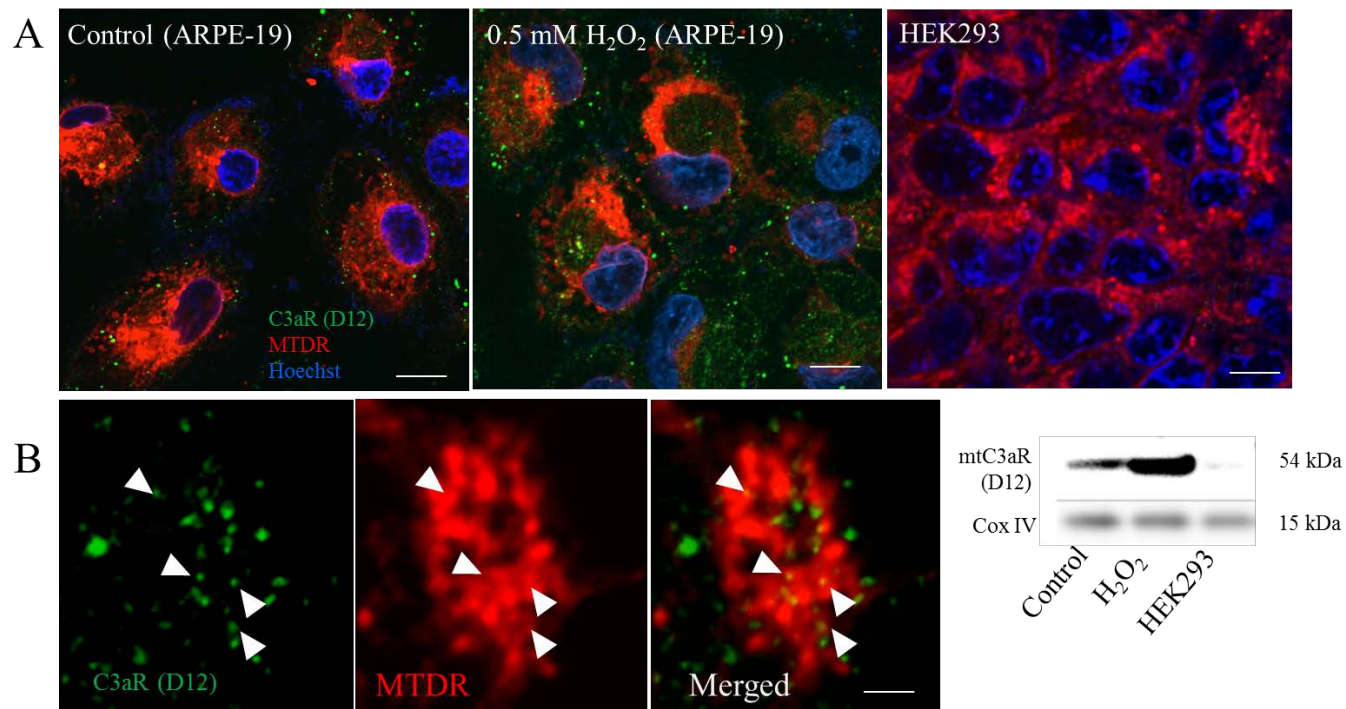

Figure S3

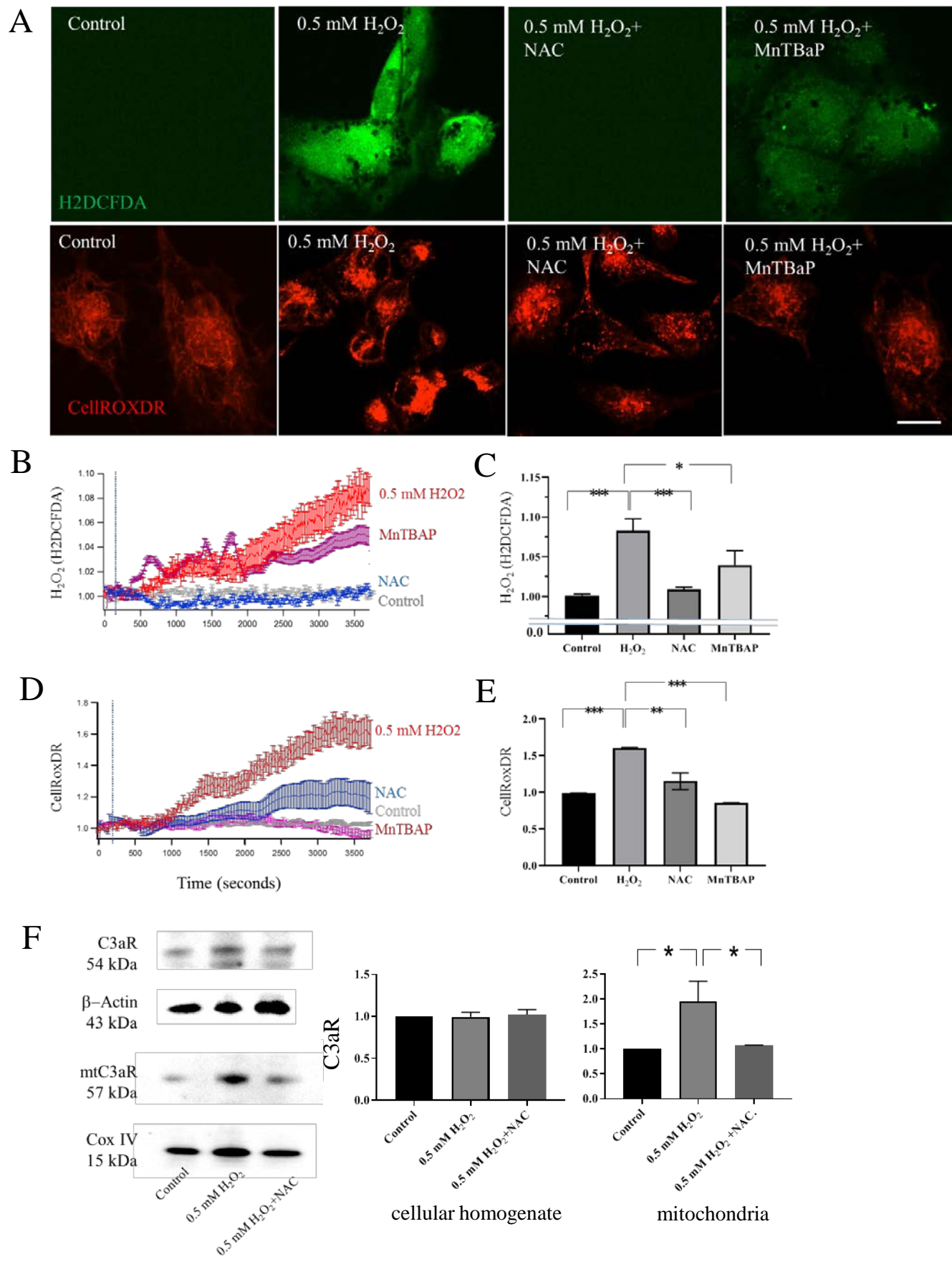

Figure S4

A

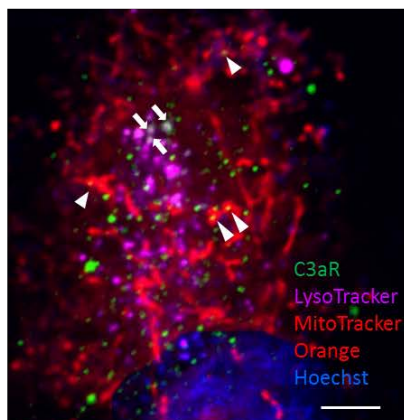

B

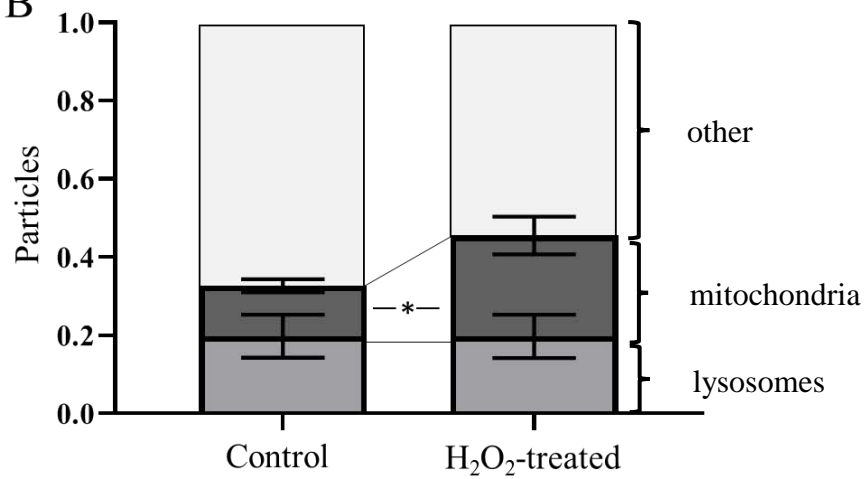

Figure S5

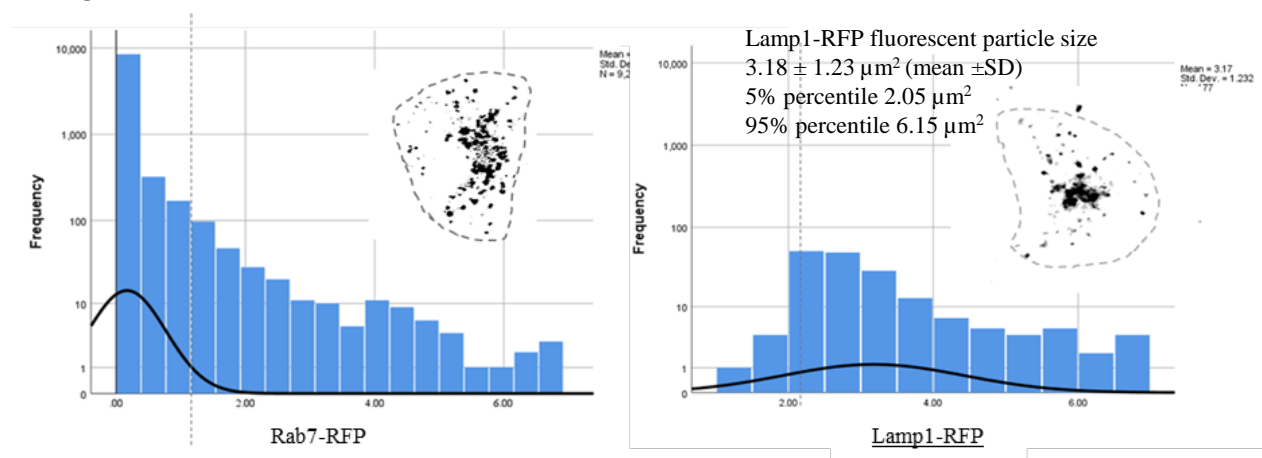

Figure S6

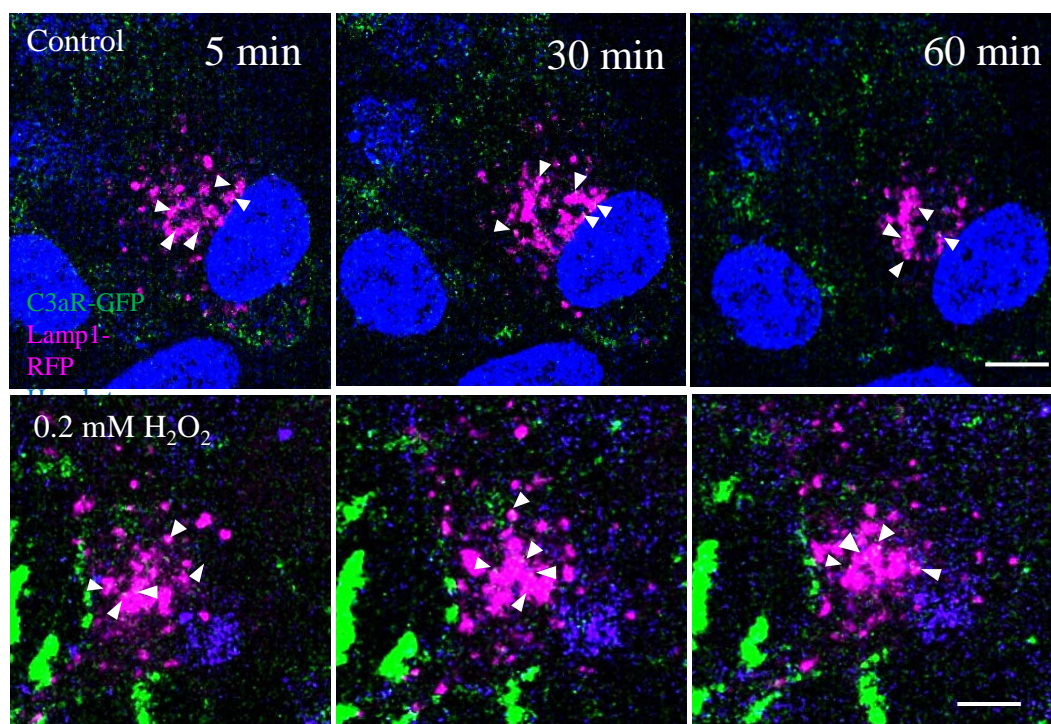

Figure S7

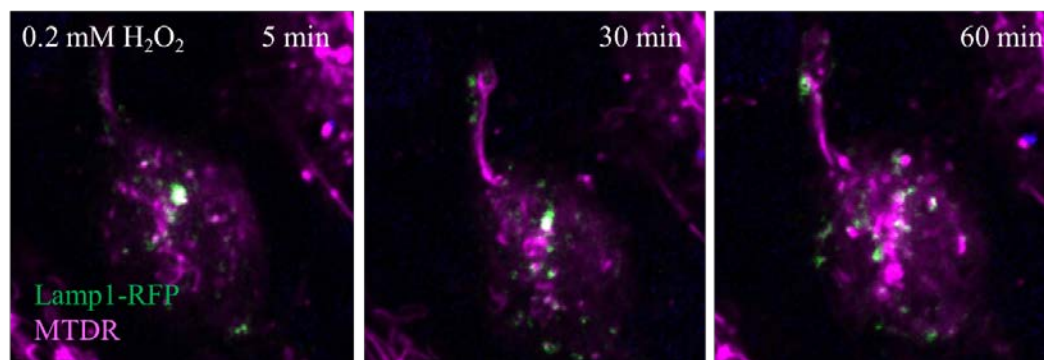

Supplement: Supplementary file 1 [file Data_Sheet_2.PDF]
