# Supplementary material for: Mitochondrial C3a Receptor Activation in Oxidatively Stressed Epithelial Cells Reduces Mitochondrial Respiration and Metabolism
Source: Front Immunol. 2021 Mar 5;12:628062. doi: 10.3389/fimmu.2021.628062 (PMC7973370; doi:10.3389/fimmu.2021.628062)
Supplement: Supplementary file 2 [file Data_Sheet_1.pdf]

Supplemental Figure Legends

Figure S1. Colocalization analysis of C3aR immunoreactive material and mitochondria using JACoP plugin in NIH ImageJ. This analysis provides an important control for Figure 1. (**A**, left panel) The cross correlation coefficent (CCF) for 60x images from MitoTracker Deep Red (MTDR) labeled mitochondria (red channel) and C3aR particle (green channel) was obtained. CCF analysis was performed by shifting the red channel (MTDR) in the X-direction, pixel by pixel, relative to the green channel (C3aR) and calculating the corresponding CCF. The peak of the CCF occurred when the red channel was not shifted (= 0 µm shift), indicating the perfect co-localization of the red and green channels. (**A**, right panel) To exclude the possibility that C3aR was randomly distributed, the red channel (MTDR) was flipped horizontally and the normalized CCF re-calculated. In this case, no peak was identified close to a shift of 0 µm, that is, the CCF required a strong shift (-5 µm) to reach a small and insigificant maximum. This result indicates that the colocalization observed in (**A**) was not a consequence of the random distribution of C3aR on mitochondria, but rather that the C3aR and MTDR signals were truly co-localized. (**B**) Depth analysis of C3aR-mitochondria colocalized particles. Images were captured at different depths. In middle image (= 0 µm), C3aR particles (green) were distributed on mitochondria labeled with MTDR (red). If the focal plain was shifted by +1 µm (right) and -1 µm (left), no colocalization between C3aR particles and MTDR signal was detected. Scale bar in (B) 10 µm. Imaging was performed in n=3 independent experiments.

Figure S2. A second anti-C3aR antibody (Clone D12, Santa Cruz, Dallas, TX) was used to confirm results obtained using the clone 17 antibody in Figure 1 of the main manuscript. (**A**) ARPE-19 cells, control and cells treated with H_2O_2 (0.5 mM) as well as HEK293 cells (negative

control) were immunolabeled for C3aR (green) in cells co-labeled with MTDR (red) for the identification of mitochondria and Hoechst33342 (blue) to identify the nucleus. C3aR particles were distributed on mitochondria and the other areas in control, cells (left), with 0.5 mM H_2O_2 treatment increasing the C3aR distribution on mitochondria (middle). No C3aR positive material was identified in C3aR negative HEK293 cells (right). (**B**) High magnification images of C3aR immunolabeling on isolated mitochondria labeled with MTDR (left). Western blot analysis for C3aR was performed on mitochondrial fractions (mtC3aR and mitochondrial Cox IV protein loading control) of control and H_2O_2-treated ARPE-19 and HEK293 cells. The results showed no remarkable differences from the results described in Figure 1. Scale bars: upper 3 images in A 10 µm, in bottom images 1 µm. Imaging and Western blotting was performed on n=3 independent samples.

Figure S3. Generation of intracellular reactive oxygen species (ROS) and superoxide anions in response to H_2O_2 and scavenging effects of N-acetylcysteine (NAC) and Mn(III)tetrakis (4-benzoic acid) porphyrin (MnTBAP). Intracellular H_2O_2 levels are detected with H_2DCFDA dye (1 mM; Thermo Scientific), superoxide anion and hydroxyl radicals were detected with CellRox Deep Red (2 mM; hermo Scientific) using confocal microscopy, imaging over a 60 minute time span. (**A**) H_2O_2 treatment increases ROS (top row, 2nd panel) and superoxide anion and hydroxyl radicals (bottom row, 2nd panel). NAC and MnTBAP inhibited the generation of intracellular H_2O_2 as well as the production of superoxide anions and hydroxyl radicals level (3rd and 4th panel respectively in top and bottom row). (**B**) H_2DCFDA fluorescence changes over 60 minutes. (**C**) H_2DCFDA fluorescence levels at the 60th minute documents a significant ROS production in response to H_2O_2 exposure, which was quenched by NAC and MnTBAP. (**D**) CellRox Deep

Red (DR) fluorescence changes over 60 minutes. (**E**) CellRox DR fluorescence levels at at the 60th minute document a significant production of superoxide anions and hydroxyl radicals in response to H_2O_2 exposure, which was quenched by NAC and MnTBAP. (**F**) Western blots of cellular (β-actin control) and mitochondrial (Cox4 control) homogenates were assayed for the presence of C3aR (left image), and quantified using ImageJ. H_2O_2 triggered a 2-fold increase in C3aR immunoreactive material in the mitochondria (right chart), but not in the cellular homogenate (left chart). NAC treatment prior to addition of H_2O_2 inhibited this transfer of C3aR immunoreactive material to the mitochondria, but had no effect on total C3aR levels in the cellular homogenate. Scale bar in (A) 10 μm. n=3 independent imaging experiments with multiple cells each, one-way ANOVA, T-test. n=3 samples for Western blotting, Kruskall-Wallis test, Mann Whitney *U*-test. * $P<0.05$, ** $P<0.001$. ***$p<0.0001$.

Figure S4. Changes in C3aR localization on mitochondria and lysosome in response to stress. (**A**) Localization of C3aR (green) on MitoTracker Orange labeled mitochondria (red) orLysoTracker Deep Red labeled lysosome (magenta) was assessed. C3aR colocalization on mitochondria is indicated as yellow (arrowhead), on lysosome as white (arrow). (**B**) Quantitative analysis of C3aR particle distribution on mitochondria and lysosomes normalized to the total number of C3aR particles (n=7) demonstrates that oxidative stress increases mitochondrial C3aR levels, but not lysosomal. n=9 cells in 3 images. Data are plotted as mean ± SEM. Mann Whiteny *U*-test, * $p<0.05$.

Figure S5. Size distribution of organelles labeled with Rab7 and Lamp1. ARPE-19 cells were transfected with Rab7-RFP or Lamp1-RFP, and the signals characterized by confocal microscopy. Labeling was binned based on size of the labeled organelles in steps of 0.5 μm at 60x magnification, identifying two non-overlapping populations, small organelles (0.17 ±0.61 μm^2) characterized by Rab7-GFP labeling, and large ones (3.17 ±0.23 μm^2) by Lamp1, and one intermediate sized population labeled by both markers. This suggests that the small organelles labeled by Rab7-RFP represent endosomes, the larger ones labeled by Lamp1 represent lysosomes. A cutoff for organelle size of 2.05 μm^2 was chosen to represent endosomes, representing 95% of the Rab7-RFP, but only 5% of the Lamp1-RFP signal. n=3 independent images, multiple cells each. Binning and frequency distribution was performed in SPSS.

Figure S6. Live cell imaging analysis for C3aR-GFP trafficking to mitochondria. ARPE-19 cells were transiently transfected with C3aR-GFP (green) and the lysosome marker Lamp1-RFP (false colored magenta). For imaging, mitochondria were labeled with Mito-Tracker Deep Red (MTDR, red) and nuclei were identified with Hoecst (blue). Representative time lapse images of 0.2 mM H_2O_2 treated cells are indicated, allowing for analysis of C3aR-GFP and MTDR as well as C3aR-GFP and Lamp1-RFP colocalization. Quantitative analysis of colocalization of the lysosome marker Lamp1-RFP and MTDR documents an increase in co-localization over the 60 minute time course (see **Fig. 4E**). Scale bar 10 μm. Representative images of n=3 independent expeirments.

Figure S7. Live cell imaging analysis for C3aR-GFP trafficking to lyosomes. ARPE-19 cells were transiently transfected with C3aR-GFP (green) and the lysosome marker Lamp1-RFP (false colored magenta). For imaging, nuclei were identified with Hoecst (blue). (Top row) Representative time lapse images of control cells are indicated, allowing for analysis of C3aR-GFP and Lamp1-RFP colocalization. Constitutive localization of C3aR to lysosomes could be documented (arrow heads). (Bottom row) Representative time lapse images of 0.2 mM H_2O_2 treated cells. Colocalization of C3aR with lysosomes could be documented (arrow heads), but not was not affected bby oxidative stress. Quantitative analysis of colocalization of C3aR-GFP and the lysosome marker Lamp1-RFP over time, documents a lack of change (see **Fig. 4F**). Scale bar 10 μm. Representative images of n=3 independent expeirments.
